# Evaluating translocation success of wild eastern hellbenders (*Cryptobranchus alleganiensis alleganiensis*) in Blue Ridge Ecoregion streams using pre- and post-translocation home range sizes and movement metrics

**DOI:** 10.1371/journal.pone.0283377

**Published:** 2023-04-20

**Authors:** Bradley D. Nissen, Michael J. Freake, Emilly Nolan, Rebecca H. Hardman, William B. Sutton

**Affiliations:** 1 Wildlife Ecology Laboratory, Department of Agricultural and Environmental Sciences, Tennessee State University, Nashville, Tennessee, United States of America; 2 Department of Biology, Lee University, Cleveland, Tennessee, United States of America; 3 Department of Forestry, Wildlife, and Fisheries, University of Tennessee, Knoxville, Tennessee, United States of America; US Geological Survey, UNITED STATES

## Abstract

Translocations of freshwater species have become a widespread conservation strategy to mitigate the impacts of habitat fragmentation, yet they are not often rigorously monitored using animal movement data to determine their success. We demonstrate the value of monitoring pre- and post-translocation movements and home-range sizes of a fully-aquatic, benthic stream salamander, the eastern hellbender (*Cryptobranchus a*. *alleganiensis*) to determine translocation success. We studied the home range sizes, movements, and habitat use of individuals (n = 27) in two self-sustaining populations (S1 & S2) for one year, and then subsequently collected similar data from a subset of these individuals (n = 17) that were translocated into two nearby streams (T1 & T2) with dam-isolated, declining populations in the Blue Ridge Ecoregion of Tennessee. We collected 1,571 location data points (869 pre-translocation and 715 post-translocation) from four study sites, and evaluated effects of mass, sex, and pre-translocation home range size/sedentariness, as well as habitat covariates on home range size and movements. Hellbender home range sizes increased from pre-translocation estimates at both sites, but response depended primarily on physical characteristics of release sites. Home range and fine-scale movement metrics indicated that hellbenders translocated from S1 to T1 settled in more quickly, had greater site fidelity, and smaller home ranges than hellbenders translocated from S2 to T2. Hellbender movements were influenced by cover rock size and density rather than individual characteristics. Study-long survival rates of translocated hellbenders increased from S1 to T1 (80% to 100%) and decreased from S2 to T2 (76% to 33%). Monitoring pre- and post-translocation movements was a valuable method for evaluating short-term translocation success in a freshwater environment. For future hellbender translocations, managers should prioritize selecting suitable release sites with contiguous boulder-dense areas (1–2 per m^2^), adequate prey (crayfish) densities (>1/m^2^), and habitats with low risk of predation.

## Introduction

Since the early 1900s, dams have become commonplace throughout the eastern United States [1], and have caused widespread harmful impacts on some of the most threatened and biologically diverse ecosystems in North America [2, 3]. Due to habitat fragmentation caused by dams [4], obligate freshwater species such as fish, mussels, and some amphibians are increasingly in need of wild translocations, as these species are unable to cross barriers and are more susceptible to the impacts of habitat fragmentations than terrestrial species [5–7]. For example, Kominoski et al. [2], found that dam-isolated fish populations in the southeastern United States were 28 times more likely to become extirpated than similar populations with natural flow. Additionally, small, isolated populations are subject to increased extinction risks due to inbreeding and genetic stochasticity [8], and thus benefit immensely from translocations designed to increase genetic diversity [9, 10]. As freshwater species are rapidly declining across the globe [3], conservation strategies such as translocations provide potential options to connect previously fragmented populations. Consequently, there is an urgent need to better understand the impacts of translocation programs on these vulnerable species.

Translocations, defined by the International Union for the Conservation of Nature (IUCN) [11] as “any human-mediated movement of living organisms from one area, with release in another,” are a widespread management strategy for freshwater wildlife conservation; however, they are still considered a risky endeavor and rigorous post-release monitoring is often warranted to determine project efficacy [11, 12]. Monitoring movements of translocated animals can be an informative tool to evaluate and improve translocation success rates by clarifying the establishment process of individuals [13, 14]. Immediately post-release, translocated animals must explore novel environments to evaluate suitability for survival and efficient resource exploitation [15, 16]. However, this “exploratory phase” can be costly (e.g. high energetic demands, increased predation risk, missed mating opportunities), so animals must continually weigh these risks with the benefits of exploiting already known resources (e.g. those near the release site) [17]. Due to these exploration risks, a successful translocation depends upon animals rapidly settling and establishing site fidelity [18]. In freshwater systems, the movements of wild translocated individuals can be strongly influenced by the characteristics of their native habitats [14, 19]. For example, multiple riverine-adapted fish species that were translocated to lacustrine environments (e.g., impoundments above dams) subsequently evacuated release sites and moved back into riverine environments in less than one month [19, 20]. Thus, translocation requires careful release site selection planning and baseline knowledge of the species’ spatial ecology within their native habitat [21, 22].

Often when evaluating the movements of wild translocated individuals, data are only collected *after* translocation–leaving a considerable gap in the knowledge of how translocation impacts natural behaviors [23]. In many cases, translocation success is evaluated by comparing translocated populations with resident populations [24, 25], but this may not be feasible if translocations are used to reintroduce animals to vacant or sparsely populated regions. Studying the movements, home-range sizes, and survival rates of wild animals *prior* to translocation could provide an important natural baseline for evaluating translocation success, through comparison to post-translocation metrics [26]. Furthermore, monitoring resident individuals may reveal spatial requirements and/or specific habitat preferences of the individuals, which may be useful for translocation site selection [27]. For imperiled freshwater taxa, understanding impacts of translocation is critical for successful conservation management [7].

Within North American riparian systems, hellbenders (*Cryptobranchus alleganiensis*) are presently in need of conservation and management measures including translocation [28, 29]. Hellbenders are large (up to 74 cm), fully-aquatic, long-lived (25+ yr.) salamanders that are slow to sexually mature (5–7 years) and require pristine, rocky, swift-flowing rivers and streams with high levels of dissolved oxygen to survive and reproduce [30, 31]. Historically, eastern hellbender populations (*C*. *a*. *alleganiensis*) were found in abundance throughout their geographic range which extends from southern New York to northeastern Mississippi, and west into Missouri, USA [31, 32]. However, populations have drastically declined across their range due in part to habitat fragmentation caused by dams [33–35]. Dams restrict dispersal and can genetically isolate populations in headwater streams [35]. Hellbender populations often already have minimal natural inter-drainage gene flow due to their highly sedentary nature [36] and reductions in population size and connectivity. For example, Crowhurst et al. [5] found that private alleles within eastern hellbender populations in Missouri may have once been shared among six out of eight major drainages surveyed, but they are now restricted to single populations. Dams can exacerbate this type of genetic isolation and cause extirpation by dividing hellbender populations within drainages, separating headwater populations from lower elevation “source” populations that are needed to counter genetic stochasticity [37]. This trend has held true in the streams of the Blue Ridge ecoregion, a threatened biodiversity hotspot [3, 38] where a majority of the remaining hellbender populations in the southeastern United States that are still showing signs of population recruitment have been separated from headwater populations by dams [37, 39]. Fragmentation poses a serious threat to hellbender populations and underscores the importance of evaluating translocation as a conservation strategy for wild hellbenders [5, 31, 37].

Recent studies have shown that when feasible, translocation of wild adult hellbenders is a more successful strategy compared to the release of captive-raised juveniles, based on greater survival rates and reproductive potential [25, 29]. Individual characteristics such as mass and/or age may also be potential indicators of hellbender translocation success (i.e., survival) [29, 40]. Furthermore, individual hellbenders may react differently to being translocated. For example, certain translocated wild hellbenders may exhibit “homing” behaviors, searching for their previous territories [30, 41], while others quickly settle into their new environments [25]. However, no previous studies have examined how the spatial ecology of individual hellbenders in their native streams could impact translocation success. Pre-translocation monitoring of wild hellbenders may offer valuable insight into the outcome of translocations by providing a paired experimental control set with which to compare the movements and home range sizes of recently translocated individuals [13, 25].

The purpose of our study was to utilize this strategy of comparing pre- and post-translocation movements to determine if translocation of wild hellbenders could serve as an effective conservation strategy for augmentation of declining hellbender populations in fragmented watersheds. Our primary goal was to identify whether individual and/or habitat-based characteristics influence wild hellbender movements and survival rates before and after translocation. To achieve this, our objectives were to 1) evaluate the effects of translocation on wild hellbender home range establishment and movement behaviors (e.g. sedentariness, and dispersal distances) at two translocation sites by comparing pre- and post-translocation movements and 2) evaluate *a priori* hypotheses to identify both environmental and individual characteristics associated with increased sedentariness and smaller home range sizes (i.e., successfully settling) after translocation.

We hypothesized that due to a likely “exploratory phase” [29, 41], home range sizes for hellbenders would increase after translocation, and the proportion of sedentariness (i.e., no movement between consecutive tracking sessions) would decrease from pre-translocation estimates. We also hypothesized that translocated hellbenders would maintain relatively high site fidelity to pre-selected release sites with greater boulder concentrations (i.e., >1 boulder/m^2^) given the importance of these habitats for refuge, foraging, and nesting [31, 42]. Lastly, we hypothesized that home range sizes of translocated individuals could be partially explained by the size of their home range prior to translocation, with individuals that had smaller home ranges (i.e., well-established territories) prior to translocation being more likely to increase their home range size after translocation due to homing behaviors [30]. Herein, we demonstrate how utilizing both pre- and post-translocation movement patterns can be a valuable strategy for monitoring the success of freshwater translocations. We demonstrate that wild hellbender translocations may serve as a viable conservation strategy for declining and fragmented populations, and we offer specific management recommendations which will be informative for future hellbender translocation projects throughout their geographic range.

## Methodology

### Study sites

#### Source sites

Our study was conducted in southeastern Tennessee, U.S.A, within the Blue Ridge Level III ecoregion of the southern Appalachian Mountains. Our general study area represents an important conservation area for hellbenders due to the presence of multiple recruiting populations, which may be limited in other portions of the contemporary geographic distribution [39]. Two streams served as source sites for wild hellbenders, hereafter referred to as source site 1 and 2 (S1, S2) to protect site locations from illegal poaching. Both sites were densely populated with healthy hellbenders of all age classes [39]. Source site 1 was a montane headwater stream with relatively high concentrations of large cobble and boulders (S1 Fig), and swift moving water (Table 1). Source site 2 (Table 1) was a 500 m section of a large river, located ~5 km downstream of a hydro-electric dam, with long stretches of metamorphic sandstone and siltstone pebble and gravel beds, interspersed with large boulders and bedrock shelves (S2 Fig) [39].

**Table 1:** Study site characteristics.

Site	Stream Order ^†^	Percent Forest in Watershed^‡^	Mean Elevation (MASL)^§^	Stream Gradient (m/km)	Mean stream width (m)
**S1**	4^th^	96.6%	485	10.07	14
**T1**	3^rd^	98.8%	460	21	8.5
**S2**	6^th^	83%	220	1.27	80
**T2**	5^th^	96%	252	2.15	20

S1 & S2 = Source Sites 1 & 2 and T1 & T2 = Translocation Sites 1 & 2.

^†^–Stream order is defined following Strahler method (1957) [43].;

^‡^–Land cover data taken from the National Land Cover database (2016);

^§^–Elevation given as meters above sea level (MASL).

#### Translocation sites

Our translocation sites, hereafter T1 and T2 (numbers correspond to the matching source site, or “cohort”), were two streams within a National Forest in Tennessee, U.S.A. We selected release sites based on their overall habitat suitability and stability (i.e., high concentrations of large substrate & pristine water quality; S1 and S2 Figs). Historically, local hellbender populations in the selected translocation sites were genetically similar to hellbenders in the source streams [37], but have declined or been extirpated in recent decades, likely in part due to isolation by dams [39].

**Fig 1:**
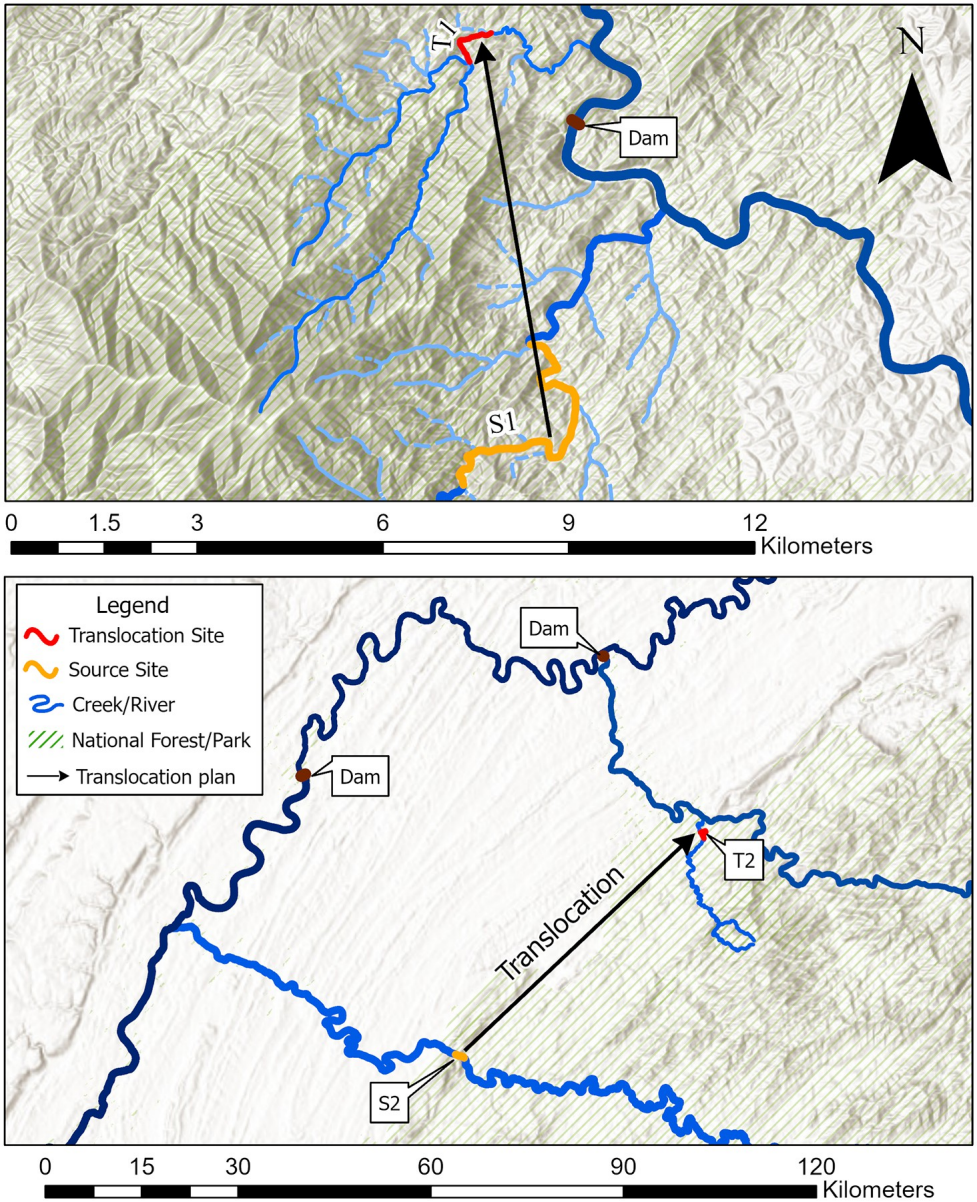
Source sites and translocation sites. Geographic representation of the relationships between both S1 and T1 (top panel) and S2 and T2 (lower panel), shown with the dams blocking hellbender migration between the source sites and translocation sites. Spatial files sourced from USGS National Map Viewer.

Translocation Site 1 was a small montane headwater stream (Table 1) composed primarily of medium to large cobble and boulders (S1 Fig) [31], and was the release site for translocated animals from S1, which is now separated from T1 by a dam installed by the Tennessee Valley Authority (TVA) (Fig 1). Recent surveys and trapping efforts have yielded very few hellbenders in the stream (<5 individuals), all of which were large (and presumably older) adults, with no signs of recruitment (M. Freake, pers comm).

Our second translocation site (T2) was comparably wider than T1 (Table 1) with disjunct clusters of relatively high amounts of large rocky substrate (i.e., cobble, boulders) and bedrock shelves (S2 Fig), surrounded by long (> 30 m), flat sections of pebble and gravel beds. The release sites on T2 were within a boulder-dense area (> 1/m^2^) and located approximately 65 km northeast of the S2 site, separated by multiple large dams (Fig 1). Recent (since 2000) surveys at this stream have yielded very few hellbenders (M. Freake, unpublished data; P. Rakes, unpublished data), and eDNA samples taken from the stream did not result in positive detections for hellbenders in 2016 [44].

### Field methods

#### Initial capture and surgery

We conducted initial hellbender sampling between 03 June—08 July 2018 at S1 and S2. We captured hellbenders via standard mask and snorkel rock-lifting surveys [45]. Upon capture, we weighed hellbenders in mesh bags with a Pesola^®^ spring scale (Pesola AG, Baar, Switzerland) and measured total length (TL) and snout-vent length (SVL) as described previously in Burgmeier et al. [36]. For individuals that were at least 150 g (n = 30), a 4 g model F1170 (pulse rate 15 ppm [708-day battery life]) radio-transmitter (Advanced Telemetry Systems, Isanti, MN) and a 12 mm passive integrated transponder (PIT) tag (Biomark, Boise, ID) were surgically implanted into the coelomic cavity on-site (performed by RHH, DVM, Ph.D.). Surgical protocols were carried out in accordance with Burgmeier et al. [36], with a few adjustments (described further in [46]. All surgery and field protocols were approved by the Institutional Animal Care and Use Committee (IACUC) at Tennessee State University (Permit #1804WS). All surgery was performed under Tricaine Methanesulfonate (MS-222) anesthesia, and all efforts were made to minimize suffering before, during, and after transmitter implantation. In addition, a blood sample was also acquired from each hellbender while under anesthesia to genetically sex the individual following protocols of Hime et al. [47]. After surgery was completed, we placed each hellbender in a recovery bin in the stream until the righting reflex was restored (approx. 30 min). We then released animals at the original point of capture and recorded locations via a Garmin^®^ Map64 handheld GPS unit (Garmin LTD., Olathe, KS, USA; accuracy ≤ 5 m).

#### Pre-translocation tracking

We radio-tracked 27 individual hellbenders (S1: n = 10; S2: n = 17) every 4 ± 2 days between June 2018 –August 2018 (summer), once a week from 01 Sept– 30 Nov 2018 (fall) and once a month from 01 Dec 2018–28 Feb 2019 (winter), which corresponded with seasonal activity levels [28]. Bi-monthly tracking resumed in spring (01 Mar 2019), until the conclusion of the first tracking session (15 May 2019). Due to S2 being downstream of a hydro-electric dam, these animals were only tracked when discharge from the hydro-electric powerhouse was reduced to safe levels for recreation, primarily every Saturday and Sunday during the summer season (June–early Oct).

We tracked hellbenders using a three element Yagi antenna with an Advanced Telemetry Systems (Isanti, MN, U.S.A) receiver (Model R410). When possible, we used a Biomark HPR Plus (Biomark, Boise, ID) PIT tag reader with a BP Plus Portable submersible antenna (Biomark, Boise, ID) to scan over the surface of cover objects and confirm the precise location of each animal, as conducted in Connock et al. [48]. At least once every 2–3 weeks we used a borescope camera (General Tools^®^ DCS660A, New York, NY) to visually confirm animal survival. Upon each re-location, we documented hellbender locations with a handheld GPS unit (Garmin^®^ eTrex 20 or Garmin^®^ GPSMap64). We primarily conducted tracking during daylight hours (between 07:00 and 19:00 EST), although nocturnal tracking was conducted opportunistically (~10–12%) at S1, T1, and T2. The hydroelectric power generation at S2 made nocturnal tracking unfeasible.

#### Habitat data collection

At each tracked location for each hellbender, we collected habitat data to test our hypothesis that cover object density and size would influence hellbender home range size and movements [42]. We defined “cover” as any substratum particle with at least one axis ≥ 26 cm and space underneath adequate for a hellbender refuge site [49]. We assessed cover object density by counting all available cover objects within a 2-meter radius of each hellbender location. We reported the size of used cover objects as the width (i.e., longest axis perpendicular to the maximum diameter), and measured the distance to the nearest cover object as the shortest distance (cm) between the used cover object and the nearest neighboring cover object (recorded as 0 if touching). We calculated averages for each of these habitat variables by individual and assigned each hellbender a unique value for these metrics based on the substrate characteristics used during the study.

#### Translocation

We translocated 17 hellbenders from both source sites over five discrete events in the spring and summer of 2019. We translocated individuals in small groups to monitor dispersal tendencies of the released individuals over a settling period of ~30 days [46]. The number of individuals translocated in a given event was dependent on accessibility of animals at that time as well as their survival throughout the first year of the study. We translocated 5 out of 10 total hellbenders from S1 to T1 on 15 May (n = 4) and 01 July (n = 1), and 12 out of 17 total hellbenders from S2 to T2 on 18 May (n = 6), 29 June (n = 5) and 13 July (n = 1).

#### Post-translocation tracking

Tracking was more frequent after translocation to maintain a fine-scale analysis of how individuals responded to translocation. We located hellbenders every 24 ± 4 hours for the first three days after translocation to monitor long-distance migrations and/or unusual movements, behaviors, or mortalities. After this initial intensive monitoring phase, individuals at both release sites were located every 3 ± 1 days throughout the summer and fall seasons until the first hard frost of the season (-2° C; 11 Nov 2019). We recaptured translocated hellbenders every 45 days (if accessible) to assess mass gains/losses as well as overall health [46]. During this same period, tracking continued at source sites for hellbenders that were not translocated (due to inaccessibility of animals under large rocks or ledges).

#### Prey availability

Because prey availability can influence animal movements [50, 51], we conducted post-hoc crayfish density surveys at the conclusion of the study for each of the four sites to evaluate prey availability for hellbenders, as crayfish are known to be a primary food source [52, 53]. Crayfish surveys were carried out once per site by randomly sampling a minimum of 30 sections (1.5 m^2^ areas) of runs and riffles using kick-sampling with a D-net as well as a seine, following methods from Mather and Stein [54]. We conducted all sampling in 150 m long stream reaches that were occupied by the hellbenders during this study.

### Data analysis

#### Movement metrics

We marked each cover object used by a hellbender with biodegradable flagging tape, which made it possible to identify if an individual (or other individuals) re-used the same location. To maximize precision, we calculated an average coordinate from all locations recorded at the same cover object (S1 Appendix). To explore fine-scale movement patterns of the individuals, we calculated daily-step lengths of individuals, which were defined as straight-line distances (m) between successive points using the distance function in R (v3.05; R Core Development Team 2019), divided by the number of days since the last location [28]. We reported observations of no movement between points as step-lengths of 0 m. We calculated the sedentariness of hellbenders by reporting the proportion of 0 m step-lengths (i.e., no movement) for each hellbender at a site. We also calculated mean step-length for each site using all hellbender movements at a site, excluding non-movement events.

#### Home range sizes

We calculated a linear home range (LHR) for each hellbender, which provides information about the total length of the stream section used by an individual. A LHR is defined as the distance between the two most extreme locations used by an individual contained within the stream boundaries [55]. We used the Linear Referencing tool in ArcGIS Pro (v. 2.5.2; ESRI^®^, Redmond, CA) to measure distances between individual locations along a polyline shapefile of the stream path (source: USGS National Map; http://nationalmap.gov), or the measurement tool in ArcGIS Pro when distances between the two most extreme points may have been perpendicular to the stream polyline layer (e.g. in S2).

For every hellbender with ≥ 20 locations, we also calculated 50% (i.e., core home ranges) and 95% fixed kernel utilization distributions to analyze where individuals spent time within their LHR. Because hellbenders tend to remain in the same location, which can cause problems with kernel estimations [56], we followed the general approach used by Bodinof et al. [28] and randomly distributed identical locations within a radius of ~30 cm (approximately one half the mean length of rocks used by the hellbenders in that study). To restrict the home range estimates to areas within stream boundaries, we delineated the bank edges of all study sites using a Trimble GeoXT receiver (Trimble^®^, Sunnyvale, CA; accuracy <1.5 m) and then imported the paths as shapefiles for minor editing in ArcGIS Pro (v. 2.5.2; ESRI^®^, Redmond, CA).

In sinuous, linearly oriented systems such as rivers and coastlines, traditional home range kernel density estimates (KDEs) based on geographic coordinates (i.e. latitude & longitude) generally fail to exclude unusable habitat for aquatic species (i.e. land) [57]. Therefore, we generated “permissible home range estimates” (PHREs) to exclude unused areas (i.e., land) in kernel estimates by transforming hellbender locations into landscape relevant positions using natural features (e.g. distance from shore, distance along a river). With this technique, landscape-based position metrics are used to form a similar evaluation grid as a geographic coordinate grid, but with axes defined by landscape-based variables instead of latitude or longitude [57]. We then generated bivariate fixed kernel densities (“ks” package in R version 3.6.3) based on “landscape space” with this grid, and then transformed the densities back into geographic space to define the home range within accessible areas (i.e. within the stream boundaries) [57, 58].

Finally, we reported size estimates using minimum convex polygons (MCPs) to make the results of this study comparable to previous studies that have utilized these methods [25, 36]. MCPs were calculated as convex hulls that contained all locations [59], using the minimum bounding geometry tool in ArcGIS Pro (2.5.0, Esri, Redlands, CA). These MCPs were clipped to exclude areas outside the stream, using similar methodology to Ross et al. [60].

#### Spatial modeling

We conducted all statistical analyses in R (v 4.1.0; R Development Core Team 2021) using both generalized linear models (GLMs) and generalized liner mixed models (GLMMs). Prior to development of models that evaluated individual and habitat-based effects on spatial movements and home-ranges, we compared pre- and post-translocation home range estimates (LHRs, PHREs) and movement metrics (e.g. sedentariness, total distance traveled) by site, using a one-way analysis of variance (α = 0.05), followed by a Tukey’s test (“stats” package in R version 4.1.0) to evaluate pairwise post-hoc differences.

We conducted four separate analyses to better understand, 1) effects of individual and habitat covariates on fine-scale (i.e. daily) hellbender movement metrics, 2) effects of those same covariates on yearly home range sizes, 3) effects of a hellbender’s yearly home range size and movement patterns prior to translocation on fine-scale movement patterns after translocation, and 4) effects of those same pre-translocation metrics on yearly home range size post-translocation. Analyses 3 and 4 were designed to evaluate the hypothesis that home range sizes before translocation could have an inverse relationship with home range sizes and movement behaviors after translocation (i.e. individuals with well-established, small home ranges in source sites may be more likely to make large dispersals after translocations). The sampling unit for all models tested was individual hellbenders within a site, meaning that in the first two analyses translocated hellbenders were represented twice in the data set, therefore a random effect for individual was added to these analyses to avoid violating the model assumption of sample independence.

#### Model development

We developed 8 *a-priori* GLMMs for the first two analyses (1 & 2) and 14 *a-priori* GLMs for the second two analyses (3 & 4) (S1 Table) to test effects of individual covariates (mass, sex, and pre-translocation home range size/sedentariness) and habitat covariates (site, average cover rock size, and average cover rock density) on hellbender home range sizes and movement behaviors. Correlations were evaluated among all explanatory variables and the least biologically relevant variables were removed if correlation was > 0.70. As a result of this screening, “distance to nearest cover object” was removed from the set of habitat variables, as this was negatively correlated (r = -0.73) with cover object density, which we considered the more biologically relevant variable. All numerical covariates (average cover rock size, cover rock densities, mass, etc.) were scaled and centered prior to modeling. For analyses 1 & 2, we included an interactive term of cohort and translocation status (Trans) to evaluate the effects of translocation on hellbender spatial ecology for each cohort. In similar models for analyses 3 & 4, this covariate (Cohort*Trans) was replaced by cohort alone. Individual sex and mass were considered because previous studies have shown that hellbender spatial ecology can vary by sex [36] and mass [40]. We also evaluated models that treated habitat variables as nested effects within translocation status to account for the possibility that these covariates differed between source and translocation sites.

#### Model fitting and validation

We fitted models for all analyses using maximum likelihood with the “stats” package and “lme4” package (v.1.1–27.1) in R (version 4.1.0) called through RStudio (version 2022.02.3). For the response variable sedentariness (analyses 1 and 3), GLMMs (analysis 1), and GLMs (analysis 3) were fit to a binomial distribution (link = logit), using a two-column matrix containing the number of “successes” (i.e. non-movements) and the number of “failures” (i.e. movements) for each individual which permits model fit to be weighted based on the number of tracking sessions for each individual [61]. For the response variable LHR (analyses 2 & 4), the LHRs were log-transformed prior to modeling, whereas generalized linear models were fit to a gamma distribution (link = inverse), which allowed the models to estimate a gamma dispersion parameter. Akaike’s Information Criterion adjusted for small sample size (AIC_c_) [62] was used to rank the fit of models generated with the AICcModAvg package in RStudio [63]. Akaike’s model weights (ω) were also calculated within this package and interpreted as the probability that each model is the model with greatest support for the sampling situation considered [62]. Inference was based on all models in the candidate set that were within a cumulative model weight of 0.9 and had greater support than the null model. Parameters within the supported models were considered well-supported if the confidence interval for the effect size did not overlap zero. We used K-fold cross-validation to evaluate the predictive ability of the top-ranked models in each of these analyses by randomly splitting the data into two groups (training/testing; 70:30 ratio) based on sampling sizes over five separate iterations [64]. We also calculated model efficiency to indicate an overall goodness of fit by comparing the model predictions to the mean of all observed values (S2 Appendix) [65].

## Results

We collected and surgically implanted transmitters in 30 total hellbenders from S1 (5 females, 7 males, 1 unknown) and S2 (9 females, 8 males). A total of 1,571 (849 resident, 722 translocated) hellbender locations were collected from the remaining 27 individuals between 03 June 2018 to 03 May 2020. The average number (± SE) of radio-tracked locations per individual at a site was 36 ± 3 (range 9–85). We observed 16 mortalities (including three that occurred one day after surgery), and three animals were right-censored because of the transmitter signal disappearing from the study site (n = 1) or the transmitter signal remaining in the same location for >1 year without the ability to confirm if the animal was alive (n = 2) (Table 2). Study-long survival rates of hellbenders (excluding mortalities that occurred one day after surgery) were 80% and 76% at S1 and S2, respectively and 100% and 33% at T1 and T2, respectively. Predation by river otters (*Lutra canadensis*) was the primary cause of mortalities at T2 (Table 2).

**Table 2:** Individual metrics and fates.

ID	Source Site	Sex	Capture date	Trans. date	Initial SVL (cm)	Initial TL (cm)	Orig. Mass (g)	Trans. Mass (g)	Known Days Alive	Fate at end of study
1	S1	U	06/03/18		24.4	37.4	292		1	Mortality†‡—S
2	S1	M	06/03/18		26.6	40.1	288		1	Mortality†‡—S
3	S1	F	06/03/18		19	28.9	150		1	Mortality†‡—S
4	S1	M	06/04/18	05/15/19	20.6	30.1	195	236	799	Alive
5	S1	F	06/05/18		25.6	39.4	440		304	Mortality§—F
6	S1	F	06/06/18		28	44.5	500		386	Mortality‡§—H
7	S1	M	06/13/18	05/15/19	18.4	29.2	160	250	790	Alive
8	S1	M	06/13/18		19.8	30.7	180		689	Alive
9	S1	M	06/13/18	07/01/19	20	31	165	249	786	Alive●
10	S1	M	06/14/18	05/15/19	25.5	38	335	350	785	Unknown¶
11	S1	M	06/14/18		25	38.4	320		347	Censored¶
12	S1	F	06/15/18	05/15/19		30.5	177	213	788	Alive●
13	S1	F	06/15/18		29.1	45.8	566		206	Censored¶
1	S2	M	06/16/18	06/29/19	24.5	36.3	300	305	687	Alive
2	S2	F	06/16/18	05/18/19	22.4	34.1	220	253	370	Mortality‡§□—P
3	S2	F	06/16/18	05/18/19	23.1	36.1	279	310	469	Mortality‡□—P
4	S2	M	06/16/18	06/29/19	26.7	40.4	310	300	395	Mortality□—U
5	S2	M	06/16/18		24.5	35.4	300		126	Censored
6	S2	F	06/16/18		23.4	37.9	270		43	Mortality□—U
7	S2	F	06/16/18	06/29/19	24.4	36.8	260	265	687	Alive
8	S2	M	06/23/18	07/13/19	25.8	37.6	280	270	462	Mortality§—P
9	S2	F	06/23/18		24.4	37.5	300		119	Mortality§—P
10	S2	F	06/23/18	06/29/19	23.2	33.6	235	260	431	Mortality□—U
11	S2	M	06/23/18		23.3	35.5	260		413	Alive
12	S2	F	06/23/18		22.8	34.6	250		105	Mortality§—P
13	S2	M	06/23/18	06/29/19	24.1	37.5	270	248	427	Mortality‡§□—P
14	S2	F	07/08/18	05/18/19	24.5	37.0	280	263	665	Alive
15	S2	M	07/08/18	05/18/19	24.2	36.0	298	288	665	Alive
16	S2	F	07/08/18	05/18/19	20.5	32.5	205	250	447	Mortality§—P
17	S2	M	07/08/18	05/18/19	25.7	38.9	350	344	416	Mortality‡§□—P

SVL = Snout vent length. TL = Total length. Orig. Mass = Mass at initial capture. Trans. Mass = Mass at translocation. Alive = individual was confirmed alive. Mortality = hellbender confirmed or presumed dead. Unknown = Status of the hellbender was unable to be confirmed. Censored = transmitter signal was lost or animal did not move in > 1 year.

^†^ = Dead within 7 days post release.

^‡^ = Body or partial remains (e.g. bones) found.

^§^ = Tracked onto land.

^¶^ = No movement detected > 1 year.

^□^ = Transmitter recovered.

^●^ = new transmitter surgically implanted.

H—Human-caused mortality.

F—Probable flood-caused mortality (tracked to debris).

P—Probable Predation event (otters suspected).

S—Probable surgery-caused mortality.

U—Unknown cause of death.

Translocated individuals recaptured for health checks at T1 (n = 4) gained an average (± SE) of 57 ± 32 g in subsequent recaptures, whereas translocated individuals that were recaptured at T2 (n = 6) lost an average mass (± SE) of 22 ± 7 g. Average crayfish densities (± SE) ranged from 0.41 ± 0.2 (S1) to 1.9 ± 0.4 (T1) crayfish/m^2^. We observed greater densities at T1 and S2 (1.92 ± 0.4 & 0.62 ± 0.1 crayfish/m^2^, respectively), with lower densities observed at T2 and S1 (0.42 ± 0.15 & 0.41 ± 0.2 crayfish/m^2^, respectively).

### Movement metrics

#### Sedentariness

**Fig 2:**
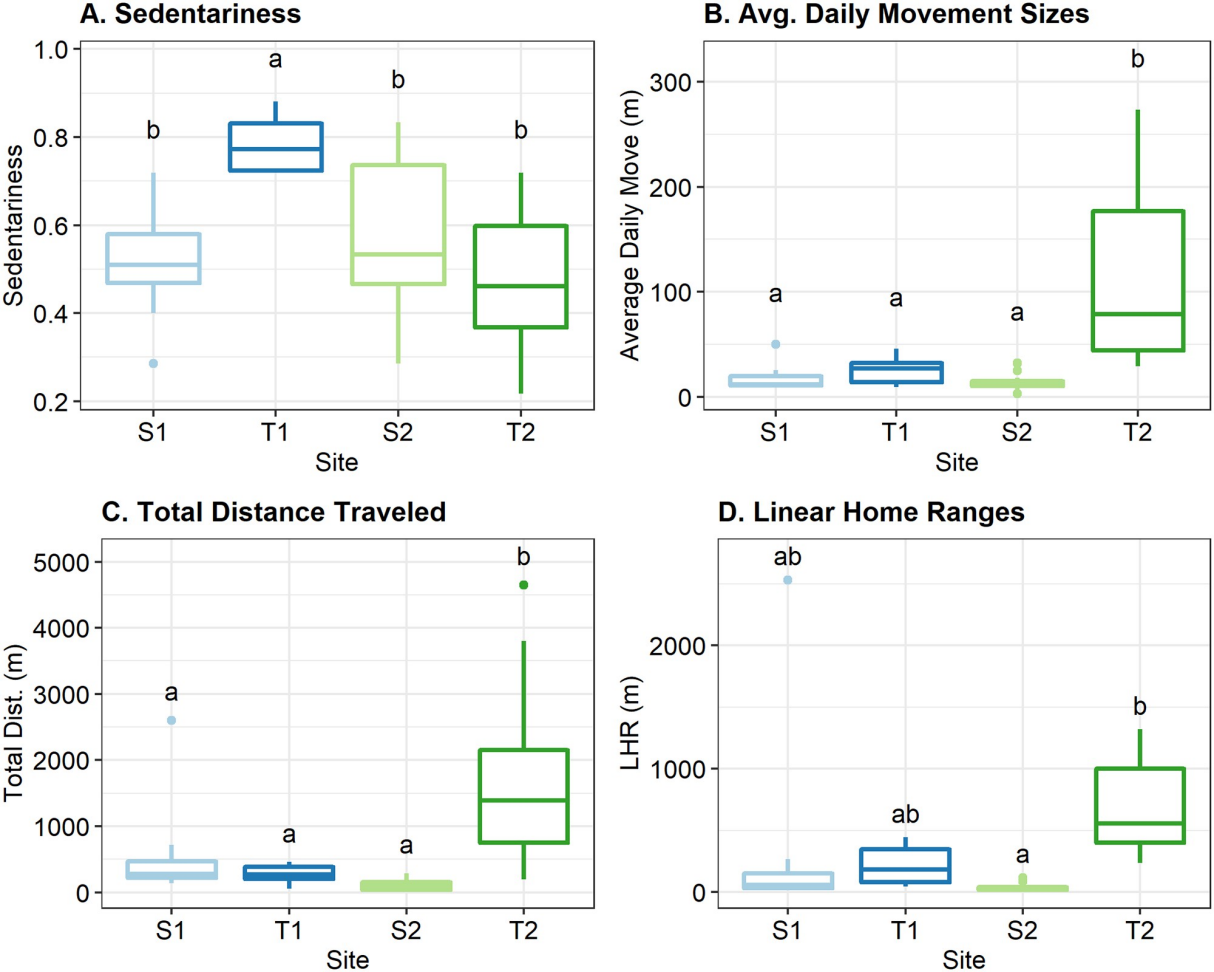
Spatial metrics by site. Boxplots displaying (A) the distribution of sedentariness proportions, (B) average daily movement sizes, (C) total distances traveled, and (D) linear home range sizes for eastern hellbenders in this study, grouped by cohort (blue = cohort 1 [S1 & T1], green = cohort 2 [S2 & T2]; light colors = source sites, dark colors = translocation sites). Letters above the boxplots indicate results of a Tukey’s *post-hoc* test to test for significant differences between the means, with different letters indicating there is a significant difference (*p <* 0.05).

Hellbenders translocated from S1 to T1 were highly sedentary and moved locations only 19% of the time. The average sedentariness rate at T1 (0.79; Table 3) was significantly greater than at any other site (*p* < 0.05; Fig 2). Hellbender movement periods at T1 were brief, lasting between 2–10 days and then individuals settled again in a new location, with all translocated individuals at T1 moving less than 20 times ($\overset{-}{X}=12$) throughout the year-long tracking period (S2 Table). By comparison, pre-translocation hellbenders at S1 (n = 10) moved an average of 25 times throughout the first year of the study, or 40% of the time between tracking sessions (S2 Table).

**Table 3:** Movement metrics by site.

Site	N	Locations	Avg. locations/Ind.	Moves	Avg. movement (m)	Avg. daily movement (m)	Total dist. traveled (m)	Avg. total dist. by Ind. (m)	Avg. sedentary
*S1*	10	520	52 ± 4	251	21.6 ± 3.08*	3.6 ± 0.4*	2948*	327.5 ± 60*	0.52 ± .04
*T1*	5	276	55 ± 3	58	25.9 ± 5.29	8.2 ± 1.5	1374	274.8 ± 70	0.79 ± .03
*S2*	17	329	19 ± 2	134	15.1 ± 1.62	3.0 ± 0.5	1809	106.4 ± 19	0.59 ± .04
*T2*	12	446	37 ± 5	213	103.2 ± 10.3	41.1 ± 4.8	21049	1754.1 ± 395	0.48 ± .04
***All Sites***	**27**	**1571**	**36 ± 3**	**656**	**42.7 ± 8.95**	**15.9 ± 3.91**	**29781**	**676.8 ± 156**	**0.55 ± .03**

Averages are reported with standard errors.

* = Outlier removed.

Hellbenders translocated to T2 (n = 12) were highly mobile, with most individuals moving locations between tracking sessions more than 50% of the time ($\overset{-}{X}$ sedentariness = 0.48; Table 3). By the conclusion of the study, 5/12 individuals at T2 had established sedentariness levels above 0.50, with levels for all hellbenders ranging from 0.21 to 0.71 (S3 Table). On average, sedentariness proportions of hellbenders at T2 decreased after translocation, but were not significantly different than pre-translocation levels at S2 (*p* = 0.32; S3 Table, Fig 2).

#### Movement distances

**Fig 3:**
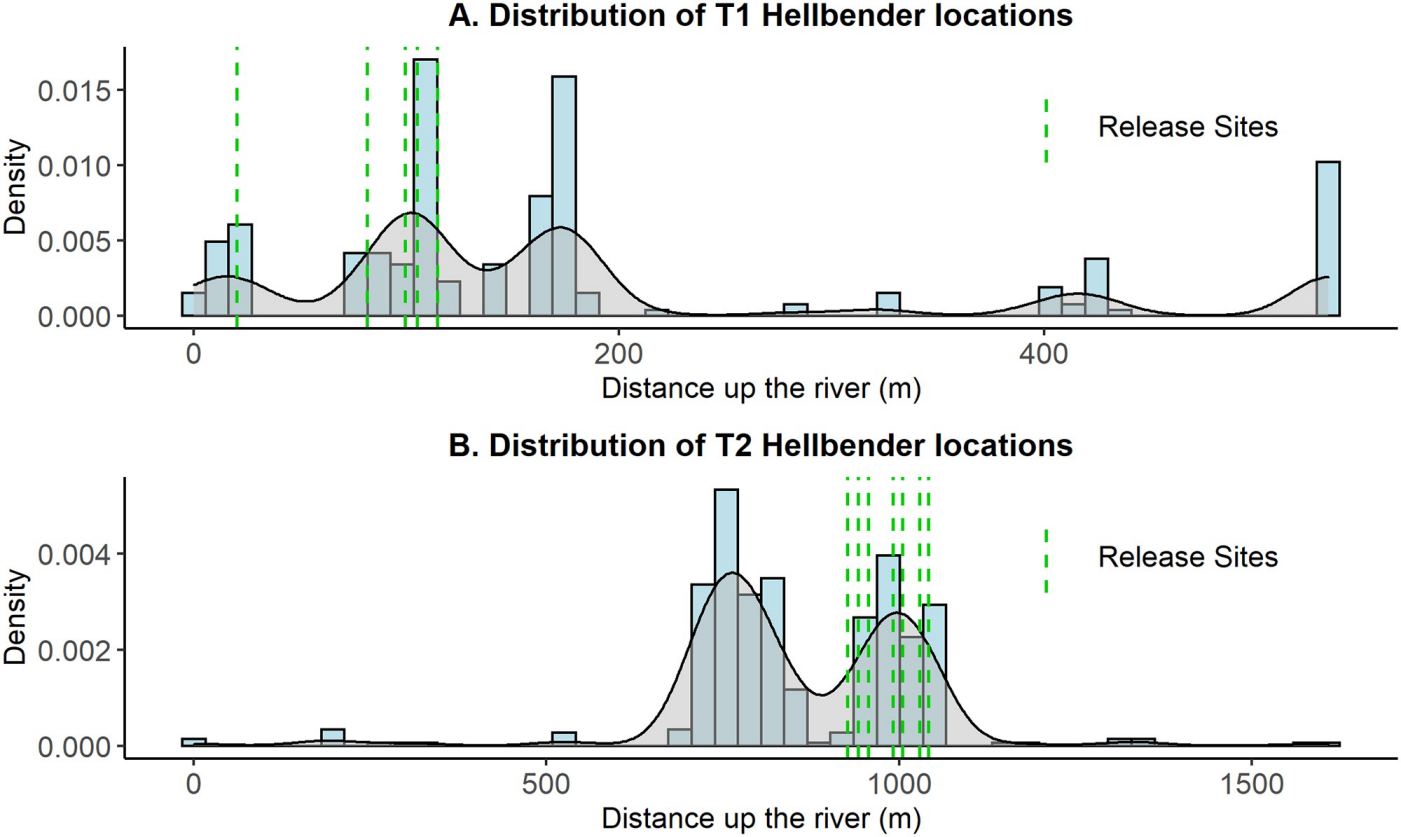
Distribution of hellbender locations at translocation sites. Histograms with density curves of all hellbender locations along the river at both T1 (A) and T2 (B) shown in relation to the release sites (dotted green lines). The most downstream hellbender location recorded during the study at each site was assigned a value of 0, and all other locations were a subsequent “distance up the river” (in meters) from that point. The more frequently that hellbenders were tracked to a certain location along the river, the greater the area under the density curve at that location.

**Fig 4:**
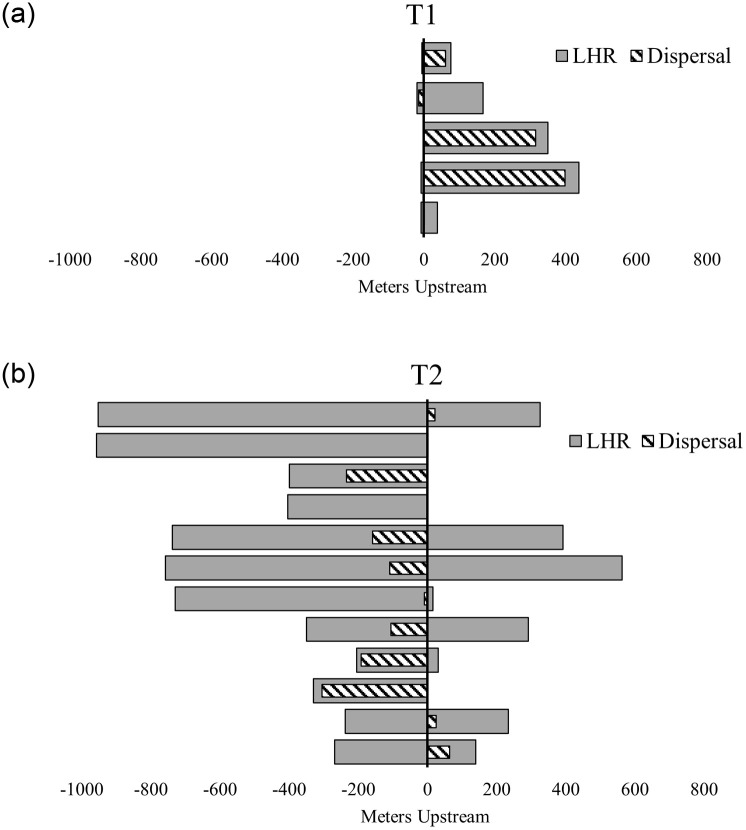
Linear home ranges and dispersal directions. Individual hellbender linear home ranges (grey) shown in relation to release sites (0 axis) and compared to the dispersal directions of these same individuals (striped). Dispersal here is defined as the distance traveled from the release site to the center of their established core home range. Each horizontal bar on the y-axis represents an individual translocated hellbender. The values along the x-axis represent the distance traveled along the stream path. Movements downstream from release sites are given negative values.

Almost all translocated hellbenders at T1 (4/5) spent the summer season (May 15 –Aug 15) rarely moving or making only small moves (<10 m/day), remaining within 50 meters of the release site (Figs 3 and 4 and S3 Fig; S2 Table). Excluding non-movements, translocated hellbenders at T1 moved an average (± SE) of 25.9 ± 5.3 m between tracking sessions or 8.2 ± 1.5 m/day (Table 3). Average step-length for translocated hellbenders increased from their respective pre-translocation levels by an average of 12 meters.

Nine out of ten resident hellbenders at S1 made daily movements that were also relatively small ($\overset{-}{X}=3.6\;m/day$) and not significantly different from the average daily movement sizes of translocated hellbenders at T1 (p = 0.98; Fig 2). All remained within the same areas of the stream the entire year except one male at S1 that dispersed upstream over 2.5 km. Only three hellbenders at S1 made moves between sequential locations that were greater than 50 m (n = 6), and this occurred only twice for each individual. Four out of six of these large moves occurred between September to October (breeding season), and all these movements were less than 20 m/day.

All translocated hellbenders at T2 made at least one very large move (>100 m) within the first two months of translocation. All translocated hellbenders at T2 significantly increased their average movement size ($\overset{-}{X}=103\;m$) from pre-translocation rates ($\overset{-}{X}=15\;m$, *p* <0.001), with an average increase of 97 meters. By comparison, all pre-translocation hellbenders in S2 exhibited high site fidelity and averaged moves of only 15 meters during the pre-release year (S3 Table). The few moves at S2 by resident hellbenders that were greater than 50 meters (n = 4) occurred during autumn (Oct.) and were made by three different individuals, two of which moved unidirectionally, and one that moved to a location and then returned to the original area in two successive large moves.

#### Dispersal

Hellbenders at T1 dispersed less than 100 m from release sites during the first months of the study (Fig 4), and the majority (3/5) traveled less than 300 meters in total (Fig 3 and S3 Fig; S2 Table). The total distance traveled by hellbenders generally decreased post-translocation by 44 meters on average (*p* = 0.92; Table 3 and S2 Table).

All released hellbenders at T2 moved over 100 meters downstream within the first month, and multiple hellbenders at T2 moved over a total of a kilometer downstream (Figs 2 and 3). In contrast, the average total distance traveled by resident individuals in S2 was just over 100 meters (Table 3), significantly less than translocated hellbenders at T2 (*p <* 0.001; Fig 2). The average total distance traveled by translocated individuals ($\overset{-}{X}=1754\;m$; S3 Table) increased by ~1.6 km from the average total distance traveled by these same individuals prior to translocation ($\overset{-}{X}=106\;m$; S3 Table). The distances traveled for hellbenders at T2 were significantly greater than the total distances traveled by hellbenders at any other site in this study (*p* < 0.01; Fig 2). These individuals did not settle in those extreme locations for more than a few tracking sessions, and instead returned to settle near release sites (Fig 3 and S4 Fig).

### Home ranges

On average, LHRs for hellbenders translocated to T1 ($\overset{-}{X}=222\;m$; Table 4) increased by ~5-fold (range: 1.1—18x; *p* = 0.96) from their pre-translocation ranges ($\overset{-}{X}$ = 103 m; S3 Table, 4), resulting in an average increase of 135 m (range: 3.6–421 m). Mean PHRE estimates for translocated hellbenders at T1 (50% core area $\overset{-}{X}=241$ m^2^; 95% area $\overset{-}{X}=1007$ m^2^) were larger, but not significantly different (p = 0.98), from individuals at S1 (50% core area $\overset{-}{X}=71$ m^2^; 95% area $\overset{-}{X}=350$ m^2^; n = 9). Hellbenders at T1 established core home ranges near release sites, or short distances upstream (Figs 3 and 4 and S3 Fig).

**Table 4:** Average home range estimates by site.

Site	Avg. LHR (m)	Avg. MCP (m^2^)	Avg. 50% PHRE (m^2^)	Avg. 95% PHRE (m^2^)
*S1*	87 ± 27*	399 ± 77*	71 ± 30*	350 ± 109*
*T1*	222 ± 77	1119 ± 355	241 ± 98	1007 ± 414
*S2*	39 ± 7	286 ± 81	32 ± 13	206 ± 76.3
*T2*	694 ± 111	7354 ± 2140	979 ± 219	5674 ± 1286
***All Sites***	**305 ± 74**	**2796 ± 846**	**587 ± 159**	**3317 ± 940**

Average home range area estimates presented with standard errors. Permissible home range estimates were only calculated for individuals with over 20 locations. LHR–Linear home range; MCP–Minimum convex polygon area; PHRE–Permissible home range estimate.

* = Outlier removed.

The LHRs at T2 were larger on average than any other site (Fig 2) and increased significantly by ~38-fold on average ($\overset{-}{X}=694\;m$; range: 3- to 142x; *p* = 0.001) from pre-translocation levels ($\overset{-}{X}=37.6\;m$). All individuals at T2 increased their LHRs from pre-translocation estimates by over 200 meters, with at least two individuals increasing their LHRs by more than a kilometer from their original LHR at S2 (range: 205–1280 m; S5 Table). Permissible home range estimates (PHREs) were calculated for all but two hellbenders at T2 (50% core area ($\overset{-}{X}=979$ m^2^; 95% area $\overset{-}{X}=5674$ m^2^; n = 10), with an average PHRE size at T2 between 30 and 27 times larger than estimates for resident hellbenders at S2, for 50% core, and 95% home range areas, respectively (Table 4).

### Spatial modeling analysis

#### Sedentariness models

In analysis 1, average cover rock size, cover object density, and an interaction between cohort and translocation status (Cohort*Trans) were the covariates included in the top sedentariness model (ω = 0.92; Table 5). Analysis 3 had a similar top model (ω = 0.92), except with cohort replacing the interaction term, since that analysis used data from only translocated hellbenders. Confidence intervals associated with beta coefficients indicated support for average rock size, density of cover objects, and an interaction between cohort and translocation status (analysis 1) or cohort (analysis 2) as drivers of sedentariness patterns (Table 6).

**Table 5:** Top-ranked spatial models.

Analysis/Top Model	AICc^a^	ΔAICc	K^b^	ModelLik^c^	ω^d^	M.E.^e^
1. Sedentariness–(All inds.)						
Environment + Status	251.91	0	7	1.00	0.92	0.27
2. LHRs–(All inds.)						
Status (Cohort*Trans)	134	0	6	1.00	0.77	-0.22
Environment + Status	136.97	3.23	8	0.20	0.15	-0.33
3. Sedentariness–(Trans. inds.)						
Environment + Status	93.55	0	4	1.00	0.92	0.71
4. LHRs–(Trans. inds.)						
Nested Rock Size	244.61	0	5	1.00	0.55	0.29*
Cohort	245.77	1.16	3	0.56	0.31	0.29*
Environment + Cohort	249.03	4.41	5	0.11	0.06	0.29*

Top-ranked models for all four spatial analyses conducted in this study, describing sedentariness and linear home range (LHR) sizes for both resident and wild translocated (i.e. all inds.) as well as for only wild translocated (Trans. inds.).

^a^Akaike’s Information Criterion adjusted for small sample sizes.

^b^Model parameters.

^c^Model likelihood.

^d^Relative model weight.

^e^Model efficiency–explained in S2 Appendix.

* = Predictions were model averaged.

**Table 6:** Sedentariness model parameters.

Model	Parameter	Estimate	Std. Error	Lower 95% CI	Upper 95% CI
1. Sedent–(All inds.)					
Environment + status	(Intercept)	0.14	0.12	-0.11	0.39
	Rock size	0.17	0.05	0.06	0.29
	Rock dens.	-0.36	0.09	-0.55	-0.17
	Cohort-2	-0.02	0.21	-0.45	0.40
	Trans-1	1.15	0.19	0.77	1.53
	Cohort-2: Trans-1	-1.01	0.29	-1.58	-0.42
3. Sedent.–(Trans inds.)					
Environment + cohort	(Intercept)	1.04	0.15	0.74	1.36
	Rock size	0.24	0.09	0.06	0.43
	Rock dens.	-0.28	0.11	-0.51	-0.06
	Cohort-2	-0.94	0.19	-1.31	-0.58

Parameter estimates for the top-ranked model describing sedentariness for both resident and wild translocated (i.e. all inds.; analysis 1) as well as for only wild translocated (Trans. inds.; analysis 3) hellbenders. Trans-1 = Translocated.

**Fig 5:**
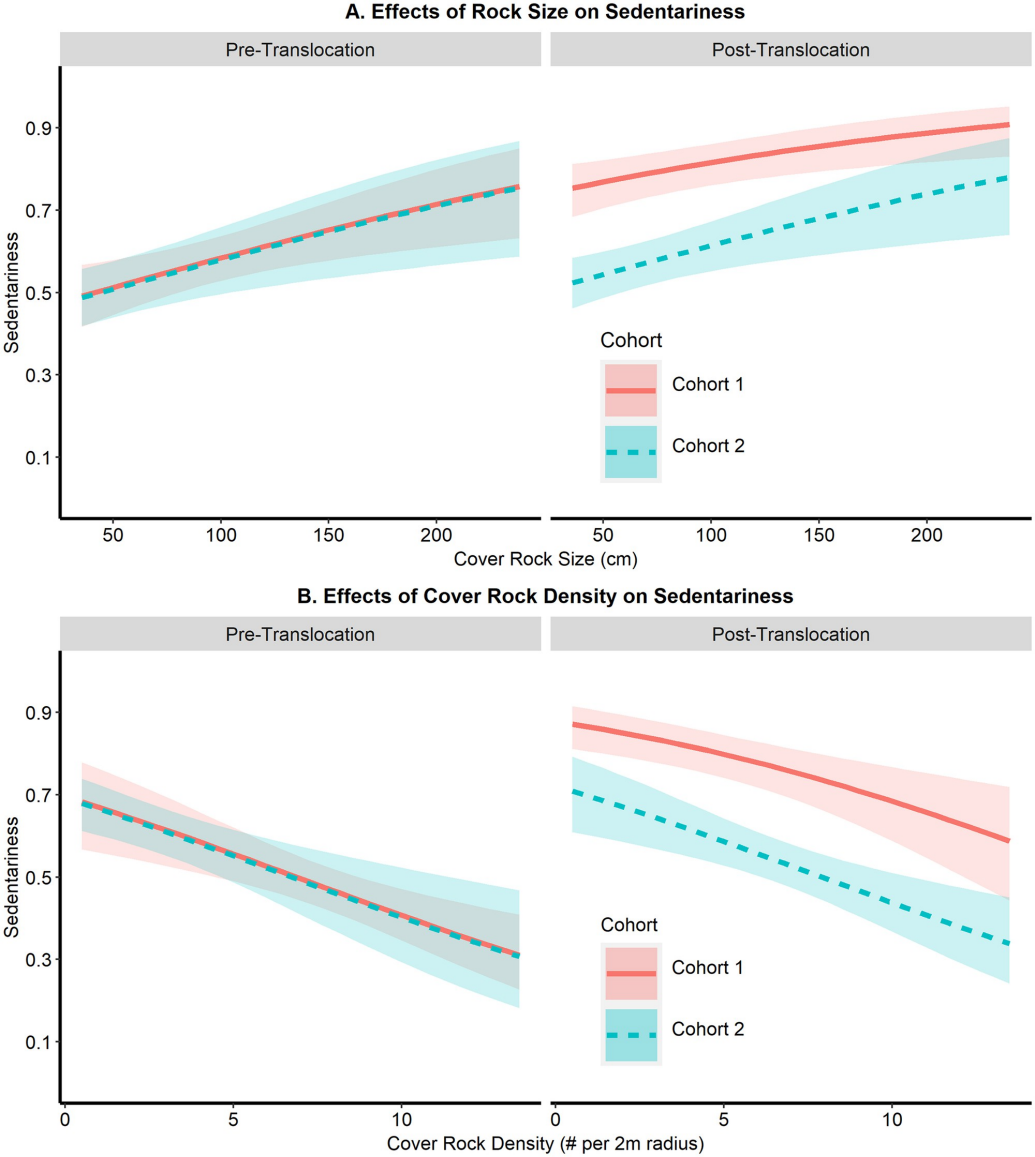
Sedentariness model predictions. Model predicted effects of (A) average cover rock size & (B) cover rock densities on the sedentariness of all eastern hellbenders in this study, grouped by translocation status and cohort. Red solid lines indicated the predicted effects on hellbenders in cohort 1, whereas blue dashed lines show predicted effects on hellbenders in cohort 2. Predicted effects are plotted for entire the range of observed rock size and rock density values across all treatments in the study, whereas shaded areas show the 95% confidence intervals for the entire range of measured rock attributes across all treatments.

Post-translocation, hellbenders in cohort 1 had a model-estimated 0.12–0.23 greater proportion of sedentariness than hellbenders in cohort 2 for all measured rock sizes (Fig 5) and a 0.16–0.25 greater proportion of sedentariness in cohort 1 than cohort 2 (post-translocation) for measured cover rock densities (Fig 5). Pre-translocation, there were no significant differences in the effects of rock attributes between cohorts. Average rock size had a positive effect on sedentariness across all translocation treatments. Models indicated that hellbender sedentariness proportions increased by 0.15–0.26 across all translocation treatments as average rock size increased from 35 cm (minimum observed) to 238 cm (maximum observed; Fig 5). Average density of cover objects had a negative effect on sedentariness. Specifically, hellbender sedentariness decreased by 0.02–0.03 for every additional cover rock available within 2 meters of their locations, and sedentariness proportions decreased by 0.29–0.37 as average rock densities increased from a mean rock density of 0.5 rocks per 2 m radius (min. observed) to 13.5 (maximum observed). Hellbender sex, mass, or pre-translocation home range size did not have a model-supported effect on sedentariness.

#### Linear home range models

An interaction effect between cohort and translocation was the only covariate in the top model for our analysis of LHRs for all individuals (analysis 2) and it carried 77% of model weight (Table 5). This model was five times more likely than the next best model (ω = 0.15), which included the same interaction effect as well as covariates for cover rock size and cover rock densities. Model results showed that both cohort and the interactive effect of cohort and translocation status had confidence intervals that did not overlap zero (Table 7). Cohort 2 as a standalone covariate had a positive effect size (0.07 ± 0.02) on hellbender LHRs, whereas the interaction between Cohort 2 and translocation had a negative effect size (-0.11 ± 0.03) on LHR (Table 7).

**Table 7:** LHR parameter estimates.

Model	Parameter	Estimate	Std. Error	Lower 95% CI	Upper 95% CI
2. LHR–(All inds.)					
Status (Cohort*Trans)	(Intercept)	0.22	0.02	0.19	0.25
	*Cohort-2	0.07	0.02	0.03	0.11
	Trans-1	-0.03	0.02	-0.07	0.01
	*Cohort-2: Trans-1	-0.11	0.03	-0.16	-0.05
4. LHR–(Trans. inds.)					
Nested Rock Size	(Intercept)	0.00352	0.00099	0.00195	0.006
	*Cohort-2	-0.00183	0.00104	-0.00431	-0.0001
	*Cohort-1: R_SIZE_	0.00442	0.002267	0.00041	0.0095
	Cohort-2: R_SIZE_	0.000485	0.000271	-0.000038	0.001

Parameter estimates for the top-ranked model describing linear home range (LHR) sizes for both resident and wild translocated (i.e. all inds.; analysis 2) as well as for only wild translocated (Trans. inds.; analysis 4) hellbenders. Trans-1 = Translocated.

*—Confidence interval does not overlap zero.

Our analysis of LHR predictors for translocated individuals (analysis 4) revealed three top models (⅀ω ≥ 0.9). These three models carried 92% of the cumulative model weight and included the nested rock size model (ω_*1*_ = 0.55), cohort model (ω_*2*_ *=* 0.31), and environment + cohort model (ω_*3*_ *=* 0.06; Table 5). The top model demonstrates that the effect of cohort, and the nested effect of cover rock size within cohort 1 had confidence intervals that did not overlap zero (S6 Table). Pre-translocation home range metrics were not supported as predictors of LHR sizes in any of the top-ranked models for translocated hellbenders (i.e. ΔAICc > 2; Table 5 and S6 Table).

#### Model validation

The five-fold cross validation of the top model for sedentariness of all hellbenders (analysis 1; Model efficiency = 0.27) and translocated hellbenders only (analysis 3; ME = 0.71) suggests that these top models were comparatively better than the mean observed value as a predictor of hellbender sedentariness. These models had low levels of error in predictive ability (17–24%; Table 5 and S7 Table). A cross validation was conducted for the top model estimating LHRs of all hellbenders, but this model was not used to make predictions, as it contained only an interaction term between categorical variables (cohort*translocation status) and had high levels of error in predictive ability (ME = -0.22; S7 Table). A cross-validation based on model-averaged predictions of the three top-models for estimating LHRs of translocated hellbenders (analysis 4) demonstrated that model efficiencies were acceptable (ME = 0.29), but relative errors for predicting LHRs were high ~55% (S7 Table), making them a poor indicator of true home range sizes and multi-model predictions.

## Discussion

By evaluating pre- and post-translocation movement data, we found that wild hellbenders increased their home range size after translocation. This supported our hypothesis that wild translocated hellbenders would have larger home ranges than residents, yet the response of hellbenders to translocation depended upon physical characteristics of release sites. Home range sizes and fine-scale movement metrics indicated that hellbenders translocated from S1 to T1 (cohort 1) settled in more quickly, had greater site fidelity, and smaller home ranges than hellbenders translocated from S2 to T2 (cohort 2). Furthermore, clear differences in survival rates between these two release sites (100% at T1; 33% at T2) emphasize the disparity in short-term translocation success between these locations. Modeling results indicate that differences in abiotic release site characteristics, such as the size and distribution of stream substrate are critical habitat components that may explain this disparity and are therefore important factors to assess during translocation site selection. Biotic site factors, such as prey density and the presence of predators (i.e. river otters) can further impact translocation success at release sites.

We found significant differences in levels of predation and survival rates at our two release sites, with river otter predation being the primary reason of high mortality rates at T2. River otters have been previously documented as predators of hellbenders [66], and our study highlights this by verifying their presence as an important source of mortality (n = 6) particularly during large movements. Exploratory movements after translocation are costly for many species [13], but especially so for highly sedentary, cryptic, territorial species such as hellbenders, which can easily exhaust their limited energy reserves or succumb to predation while exposed [40]. Less contiguous patches of boulders and/or available cover objects could be one possible explanation for the pattern of larger exploratory movements we observed for hellbenders at T2, as well as their susceptibility to predation. Although release sites at both T1 and T2 were strategically selected to contain large concentrations of boulders and bedrock shelves, areas within 200 meters of the primary release sites at T2 were primarily pebble/gravel beds and comparatively barren of large boulders or other refugia. Thus, when hellbenders made exploratory movements at T2 they traversed long stream stretches with little to no available cover objects. By comparison, cover objects at T1 were more homogenously distributed throughout the study area, which provided refugia in all sections of the stream. This may explain why hellbenders at this site were more likely to remain near the release site and expended time exploiting already known resources, rather than making costly exploratory movements up or downstream. Had there been a larger corridor with connected cover objects at T2 instead of these “islands” of suitable habitat, hellbenders might not have needed to travel such great distances (> 1 km in some cases) to encounter conspecifics, forage, or seek protection from predators (i.e. otters). Therefore, our findings suggest that selection of release sites with connected sources of refugia may dramatically minimize risk of otter depredation of translocated animals.

These findings are consistent with previous studies (e.g. [28, 42]) which found that hellbenders released into sites with larger connected areas of boulders moved shorter distances, maintained smaller home range sizes (< 50 m^2^), and were more likely to establish site fidelity than hellbenders released into areas with patchier boulder habitat. Our study found that hellbenders at T1 quickly established small home ranges near release sites, gained weight, and had higher sedentariness rates than prior to translocation–all of which indicate initial translocation success [13]. In some cases, hellbenders at T1 spent weeks under the same rock where they were released. The average step-length distances of hellbender movements at T1 (8.2 ± 1.5 m) are consistent with normal spatial ecology patterns observed for resident wild adult hellbenders by Ball (10.14 m–Males, 14.6 m–Females) [67] and translocated juveniles (1–5 m) [28]. The movement sizes of hellbenders at T1 were considerably smaller than results from other wild hellbender translocation studies ($\overset{-}{X}=55.7\;m$) [25], which may be explained by the large availability of cover objects at this site, thereby decreasing the need to move long distances to find refugia or prey. It is important to note that most of the tracking for this study was conducted during daylight hours when hellbenders are known to be less active, so our reported movement sizes and home ranges here should be considered conservative. Nonetheless, the small movement sizes and home range areas of hellbenders at T1 indicate individuals found the release habitat sites suitable and are successfully adapting to this translocation site.

By contrast, some larger exploratory movements (>1 km) made by hellbenders at T2 were greater than the longest dispersal distances reported by many other studies for both resident (e.g. 990 m–[68]; 347 m–[36]) or translocated hellbenders (550 m–[28]; >500 m [25]). Notably, most hellbenders at T2 also established core home ranges near the initial release site area, where concentrations of boulders were greater than the surrounding regions, despite their initial large dispersals. Results at both sites provided support for the hypothesis that individuals would (eventually) settle near release sites with high boulder densities. This underscores the importance of high concentrations of boulders (> 1/m^2^) for establishing site fidelity and shifting from exploring their habitat to exploiting available resources.

The availability of resources, especially prey availability (i.e. crayfish densities) could be an additional explanatory biotic factor in the differences observed between these two translocation sites, as we found considerably greater crayfish densities at T1 compared to T2. We considered evaluating prey densities only after observing significant success discrepancies between these two sites. Due to our findings, we recommend that future hellbender translocation projects take crayfish abundance into consideration when selecting release sites. Limited prey availability and the need to forage could be one explanation for the larger exploratory movements of hellbenders at T2. Previous research has also shown a strong correlation between coarse boulder habitat and crayfish densities [69], emphasizing the importance of these habitat characteristics for a successful translocation. There were also considerably greater numbers of large predators at T2 due to its larger size (5^th^ order), so hellbenders were likely competing with larger fish for prey and refugia under cover rocks [69].

Interestingly, the top-ranked sedentariness models predicted that hellbenders would decrease sedentariness with greater cover object density. Given the goal of increasing hellbender site fidelity after translocation, it may seem counterintuitive to select sites with greater cover object densities. However, this prediction could be explained by the hellbenders’ ecology and their aversion to exposure, especially for extended periods of time, as they are known to be primarily nocturnal [31, 70]. Even at night they often remain under the protection of cover objects [71]. Greater concentrations of cover objects within a small area would decrease exposure risk by allowing hellbenders to safely forage or explore an area while never straying far from the protection of an available cover object. By contrast, a hellbender sheltering under a rock with very few or no cover objects nearby might be discouraged from moving locations, thus increasing sedentariness, as the exposure risk for traveling to the next cover object may be prohibitively high. Consequently, although greater concentrations of cover objects may decrease the chances of a hellbender from remaining in the exact same location (i.e. sedentariness), it may provide greater site fidelity by offering greater protection from predators and foraging opportunities [42, 72].

In addition to evaluation of habitat characteristics important for translocations, this study also evaluated how differences in individual spatial ecology patterns or characteristics could impact translocation success. We did not detect differences in home range sizes by sex or size (i.e. mass) for resident or translocated wild adult hellbenders, which was consistent with results from other studies [25, 30]. Instead, we found that habitat characteristics at the translocation site were more important to translocation success than individual characteristics or pre-translocation home range sizes, which emphasizes the importance of prioritizing release site evaluation for translocation planning.

Although individual characteristics did not predict translocation success, studying individuals pre- and post-translocation was valuable for evaluating the impacts of translocation. This methodology allowed us to clearly distinguish the differences in how hellbenders reacted to translocation at both sites, with individuals at T2 drastically increasing their home ranges and movement sizes from pre-translocation rates, whereas hellbenders at T1 had home ranges that were very similar to pre-translocation estimates. Furthermore, this methodology, although time-intensive, allowed us to clearly evaluate the presence of behaviors such as reproduction, by using the source sites as a natural baseline. For example, when tracking hellbenders in source sites, we observed an increase in movement distances and frequency during the late summer/early autumn (i.e. Aug–Oct), which corresponds with the hellbender breeding season [30, 73]. After translocation at T1 and T2, we observed hellbenders making similarly larger moves during autumn. The comparable timing of these increased movements, especially at T1 after more than two months of highly sedentary behavior, suggests that translocated hellbenders were well-established and reacted to seasonal cues, which is promising for potential reproduction. During the breeding season, two hellbenders (male & female) were tracked to the same rock on two separate occasions at T2, indicating possible reproductive behavior. A larval hellbender was also found during the following spring very near one of the main core areas where hellbenders established home ranges, strongly suggesting that this was an offspring of translocated hellbenders (S5 Fig). These findings offer hope that wild translocated hellbenders can reproduce within the same year as being translocated if environmental conditions are suitable. Thus, collection of pre-translocation data in addition to post-translocation monitoring proved to be a valuable tactic for evaluating translocation success, and we recommend that it be considered for other translocation projects–especially when the natural spatial ecology of the species may be understudied.

This tactic of monitoring a species spatial ecology pre- and post-translocation has underscored the important impact of release site selection on translocation success for freshwater species fragmented by dams. While large dam removal in many systems is unlikely to occur in the near future due to economic and environmental concerns [74], we found that translocation around dammed systems may provide an option to increase population connectivity of isolated freshwater species within a meaningful time frame. This strategy has been used successfully for a variety of other imperiled freshwater species, including mussels [75], fish [76], and now hellbenders [46]. However, release site selection is a critical piece of a successful freshwater translocation [19] and our study highlights the value of using pre-translocation monitoring to determine release site suitability and measure translocation success. Despite their alluring potential for rapid species conservation in freshwater systems [7], translocations should always be conducted with rigorous post-release monitoring, and in conjunction with larger scale efforts to restore habitat connectivity on a long-term scale [75].

In conclusion, we found that given a suitable release site (i.e. large contiguous concentrations of boulders, high prey availability, and limited predator presence), wild translocated hellbenders could be considered initially “successful” in their new environments. This was determined by the fact that they survived, gained weight, established new home ranges with similar sizes to resident hellbenders, and possibly reproduced, all within the first year of translocation. However, if wild hellbenders are reintroduced into regions with limited prey resources, and/or patchy configurations of boulders, they may react poorly by traveling great distances, losing weight, and potentially succumbing to predation. Wild translocations should only be considered when there is a sufficiently abundant source population to withstand the loss of those individuals. Therefore, wild translocations of hellbenders could be most beneficial for regions where suitable habitat exists without healthy hellbender populations, especially if the populations have been extirpated or isolated from larger populations because of dams. Given the duration of this study and ever-increasing threats to hellbender populations, it would be worthwhile to focus future research on how successfully translocated hellbenders may continue to influence the long-term (> 10 yr.) viability of these augmented populations by studying their genetic variation and fitness levels.

## Supporting information

S1 Fig Plot of substrate distribution in S1 & T1. Comparison of the distributions of substrate sizes for each stream in the study, comparing source and translocation sites by watershed. Pebble counts were conducted at all streams following methods standardized for quantifying hellbender habitat [77].
(TIF)

S2 Fig Plot of substrate distribution in S2 & T2. Comparison of the distributions of substrate sizes for each stream in the study, comparing source and translocation sites by watershed. Pebble counts were conducted at all streams following methods standardized for quantifying hellbender habitat [77].
(TIF)

S3 Fig Map of hellbender movements at T1. Map displaying individual hellbender locations and movement directions at translocation site 1 from 2019–2020. Hellbender locations are given in circles (females) or triangles (males), and each color represents locations of a different hellbender. Colors are labeled by individual ID (i.e., 4, 7, 9, 10 or 12). Sizes of the location markers are proportional to the number of times an individual was observed at that location, with more locations having larger markers. Permissible home range estimates are used to showcase where hellbenders were most frequently observed (inset map) during the year-long post-translocation season (2019–2020). The orange line depicts the linear home range (LHR) of individual #10 –with labels indicating distances traveled from the release site. Spatial imagery and files sourced from USGS National Map Viewer.
(TIF)

S4 Fig Map of hellbender movements at T2. Map displaying individual hellbender locations and movement directions at translocation site 2. Hellbender locations are given in circles (females) or triangles (males), and each color represents locations of a different hellbender. Numbered labels along the river also denote individual IDs. Sizes of the location markers are proportional to the number of times an individual was observed at that location, with more locations having larger markers. Permissible home range estimates are used to showcase where hellbenders were most frequently observed (red circles; inset maps) over the year-long 2019–2020 sampling season. The colored lines depict the linear home range (LHR) of individuals–with labels indicating individual ID and total distances traveled between the most extreme points for that individual. Spatial imagery and files sourced from USGS National Map Viewer.
(TIF)

S5 Fig Map of larval hellbender location at T2. A simple map displaying where a larval hellbender was found at T2 in relation to the areas used most frequently by the translocated hellbenders at that site.
(TIF)

S1 Table Spatial ecology models. *A priori* models representing hypotheses, model structures, and predicted effects for factors associated with the spatial ecology (i.e. movement behaviors and home range sizes) of Eastern Hellbenders. For analyses 3 and 4 (translocated individuals only), models that included translocation status as a covariate (“Trans”) were tested by replacing “Trans” with the covariate cohort. In cases of interactive terms, the interaction with “Trans” was dropped and Cohort was used alone. Random effects of individual were not included in analysis 3 and 4. *—model tested only during analysis of translocated individuals.
(DOCX)

S2 Table Individual movement metrics and habitat data. Summary statistics of movement metrics and habitat data by individual hellbender for S1-T1 cohort (S2 Table) and S2-T2 cohort (S3 Table). Post-translocation metrics (colored) are presented for all individuals that were translocated. Where applicable, averages are given with standard errors. Trans. = Translocation. Loc. = Locations. Dist. = Distance. Sedent. = Sedentariness. Dens. = Density. ♀ = Female; ♂ = Male. * = less than 15 locations.
(DOCX)

S3 Table Individual movement metrics and habitat data. Summary statistics of movement metrics and habitat data by individual hellbender for S1-T1 cohort (S2 Table) and S2-T2 cohort (S3 Table). Post-translocation metrics (colored) are presented for all individuals that were translocated. Where applicable, averages are given with standard errors. Trans. = Translocation. Loc. = Locations. Dist. = Distance. Sedent. = Sedentariness. Dens. = Density. ♀ = Female; ♂ = Male. * = less than 15 locations.
(DOCX)

S4 Table Individual home range sizes. Summary statistics of home range sizes by individual hellbender for S1-T1 cohort. Pre- and post-translocation metrics are presented for all individuals that were translocated; post-translocation rows are colored. Kernel density estimates (KDEs) and permissible home range estimates (PHREs) were only calculated for individuals with more than 20 locations at a site. Trans. = Translocation. LHR = Linear home range. MCP = Minimum convex polygon home range. ♀ = Female; ♂ = Male.
(DOCX)

S5 Table Individual home range sizes. Summary statistics of home range sizes by individual hellbender for S2-T2 cohort. Pre- and post-translocation metrics are presented for all individuals that were translocated; post-translocation rows are colored. Kernel density estimates (KDEs) and permissible home range estimates (PHREs) were only calculated for individuals with more than 20 locations at a site. Trans. = Translocation. LHR = Linear home range. MCP = Minimum convex polygon home range. ♀ = Female; ♂ = Male.
(DOCX)

S6 Table LHR parameter estimates. Parameter estimates for the top-ranked models describing linear home ranges (LHRs) for wild translocated *Cryptobranchus a*. *alleganiensis* in eastern TN, USA, 2018–2019. * = Confidence interval does not overlap zero.
(DOCX)

S7 Table Top model validation statistics. Validation statistics for top models (see Table 5) of each modeling analysis for predicting sedentariness and linear home ranges (LHR) of Eastern Hellbenders (*Cryptobranchus a*. *alleganiensis*) using *k*-fold cross-validation. Sedent. = Sedentariness. Values that are not given as a percentage here (i.e. not AB % or RE %), are in the units of the response variable (i.e. as a proportion between 0–1 for sedentariness, and meters for LHR). Formulas for these metrics (and brief explanations) are given in S2 Appendix. *—Model-averaging used for predictions.
(DOCX)

S1 Appendix Weighting formula. (DOCX)

S2 Appendix Equations for model validation metrics. (DOCX)

S1 Data Dataframe for modeling analysis. (CSV)

S2 Data R-code. Modeling code for spatial analysis.
(R) Click here for additional data file.

## References

[1] Graf W. Dam Nation: A Geographic Census of American Dams and Their Large-Scale Hydrologic Impacts. Water Resour Res. 1999;35(4):1305–11.

[2] Kominoski JS, Ruhí A, Hagler MM, Petersen K, Sabo JL, Sinha T, et al. Patterns and drivers of fish extirpations in rivers of the American Southwest and Southeast. Glob Chang Biol. 2018;24(3):1175–85. doi: 10.1111/gcb.13940 29139216

[3] Collen B, Whitton F, Dyer EE, Baillie JEM, Cumberlidge N, Darwall WRT, et al. Global patterns of freshwater species diversity, threat and endemism. Glob Ecol Biogeogr. 2014;23(1):40–51. doi: 10.1111/geb.12096 26430385 PMC4579866

[4] Hoffman R, Dunham J. Fish-Movement Ecology in High-Gradient Headwater Streams: Its Relevance to Fish Passage Restoration Through Stream Culvert Barriers. U.S. Geological Survey Open-File Report 2007–1140. 2007.

[5] Crowhurst RS, Faries KM, Collantes J, Briggler JT, Koppelman JB, Eggert LS. Genetic relationships of hellbenders in the Ozark highlands of Missouri and conservation implications for the Ozark subspecies (*Cryptobranchus alleganiensis bishopi*). Conserv Genet. 2011;12(3):637–46.

[6] McCartney M. Living with dams: Managing the environmental impacts. Water Policy. 2009;11(SUPPL. 1):121–39.

[7] Olden JD, Kennard MJ, Lawler JJ, Poff NL. Challenges and Opportunities in Implementing Managed Relocation for Conservation of Freshwater Species. Conserv Biol [Internet]. 2011;25(1):40–7. http://doi.wiley.com/10.1111/j.1523-1739.2010.01557.x 20666802 10.1111/j.1523-1739.2010.01557.x

[8] Frankham R, Bradshaw CJA, Brook BW. Genetics in conservation management: Revised recommendations for the 50/500 rules, Red List criteria and population viability analyses. Biol Conserv [Internet]. 2014;170:56–63. 10.1016/j.biocon.2013.12.036

[9] Weeks AR, Heinze D, Perrin L, Stoklosa J, Hoffmann AA, Van Rooyen A, et al. Genetic rescue increases fitness and aids rapid recovery of an endangered marsupial population. Nat Commun [Internet]. 2017;8(1):1–6. 10.1038/s41467-017-01182-3 29057865 PMC5715156

[10] Frankham R. Genetic rescue of small inbred populations: meta-analysis reveals large and consistent benefits of gene flow. Mol Ecol. 2015;24(11):2610–8. doi: 10.1111/mec.13139 25740414

[11] IUCN Species Survival Commission. Guidelines for Reintroductions and Other Conservation Translocations [Internet]. Vol. 1, Guidelines for Reintroductions and other Conservation Translocations. 2013. 72 p. https://portals.iucn.org/library/efiles/documents/2013-009.pdf

[12] George AL, Kuhajda BR, Williams JD, Cantrell MA, Rakes PL, Shute JR. Guidelines for Propagation and Translocation for Freshwater Fish Conservation. Fisheries. 2009;34(11):529–45.

[13] Berger-tal O, Saltz D. Using the movement patterns of reintroduced animals to improve reintroduction success. Curr Biol. 2014;60(4):515–26.

[14] Carpenter-Bundhoo L, Butler GL, Espinoza T, Bond NR, Bunn SE, Kennard MJ. Reservoir to river: Quantifying fine-scale fish movements after translocation. Ecol Freshw Fish. 2020;29(1):89–102.

[15] Frair J l., Merrill EH, Allen JR, Boyce MS. Know Thy Enemy: Experience Affects Elk Translocation Success in Risky Landscapes. J Wildl Manage. 2007;71(2):541–54.

[16] Berger-Tal O, Nathan J, Meron E, Saltz D. The exploration-exploitation dilemma: A multidisciplinary framework. PLoS One. 2014;9(4). doi: 10.1371/journal.pone.0095693 24756026 PMC3995763

[17] Eliassen S, Jørgensen C, Mangel M, Giske J. Exploration or exploitation: Life expectancy changes the value of learning in foraging strategies. Oikos. 2007;116(3):513–23.

[18] Le Gouar P, Mihoub JB, Sarrazin F. Dispersal and Habitat Selection: Behavioural and Spatial Constraints for Animal Translocations. In: Ewen JG, Armstrong DP, Parker KA, Seddon PJ, editors. Reintroduction Biology: Integrating Science and Management. First edit. Blackwell Publishing Ltd.; 2012. p. 138–64.

[19] Taylor AT, Peterson DL. Movement, homing, and fates of fluvial-specialist shoal bass following translocation into an impoundment. Southeast Nat. 2015;14(3):425–37.

[20] Irving DB, Modde T. Home-range fidelity and use of historic habitat by adult Colorado pikeminnow (*Ptychocheilus lucius*) in the White River, Colorado and Utah. West North Am Nat. 2000;60(1):16–25.

[21] Armstrong DP, Seddon PJ. Directions in reintroduction biology. Trends Ecol Evol. 2008;23(1):20–5. doi: 10.1016/j.tree.2007.10.003 18160175

[22] Germano JM, Bishop PJ. Suitability of amphibians and reptiles for translocation. Conserv Biol. 2009;23(1):7–15. doi: 10.1111/j.1523-1739.2008.01123.x 19143783

[23] Riedl SC, Mushinsky HR, McCoy ED. Translocation of the gopher tortoise: Difficulties associated with assessing success. Appl Herpetol. 2008;5(2):145–60.

[24] Pinter-Wollman N, Isbell LA, Hart LA. Assessing translocation outcome: Comparing behavioral and physiological aspects of translocated and resident African elephants (*Loxodonta africana*). Biol Conserv [Internet]. 2009;142(5):1116–24. 10.1016/j.biocon.2009.01.027

[25] McCallen EB, Kraus BT, Burgmeier NG, Fei S, Williams RN. Movement and Habitat Use of Eastern Hellbenders (*Cryptobranchus alleganiensis alleganiensis*) Following Population Augmentation. Herpetologica [Internet]. 2018;74(4). http://www.hljournals.org/doi/10.1655/Herpetologica-D-17-00044.1

[26] Strum SC. Measuring success in primate translocation: A baboon case study. Am J Primatol. 2005;65(2):117–40. doi: 10.1002/ajp.20103 15706585

[27] Stamps JA, Swaisgood RR. Someplace like home: Experience, habitat selection and conservation biology. Appl Anim Behav Sci. 2007;102:392–409.

[28] Bodinof CM, Briggler JT, Junge RE, Beringer J, Wanner MD, Schuette CD, et al. Postrelease Movements of Captive-Reared Ozark Hellbenders (*Cryptobranchus alleganiensis bishopi*). Herpetologica. 2012;68(2):160–73.

[29] Kraus BT, Mccallen EB, Williams RN. Evaluating the Survival of Translocated Adult and Captive-reared, Juvenile Eastern Hellbenders (*Cryptobranchus alleganiensis alleganiensis*). Herpetologica [Internet]. 2017;73(734):271–6. http://www.bioone.org/doi/full/10.1655/Herpetologica-D-16-00009

[30] Hillis RE, Bellis ED. Some Aspects of the Ecology of the Hellbender, *Cryptobranchus alleganiensis alleganiensis*, in a Pennsylvania Stream. J Herpetol. 1971;5(3–4):121–6.

[31] Nickerson MA, Mays CE. The Hellbenders: North American “Giant Salamanders”. Milwaukee Public Museum. 1973;389.

[32] Foster RL. Lessons from the past: a historical approach to conservation of the eastern hellbender salamander (*Cryptobranchus alleganiensis*). University of Buffalo, State University of New York; 2018.

[33] Burgmeier NG, Unger SD, Sutton TM, Williams RN. Population Status of the Eastern Hellbender (*Cryptobranchus alleganiensis alleganiensis*) in Indiana. J Herpetol. 2011;45(2):195–201. 10.7589/0090-3558-47.4.836 22102654

[34] Foster RL, Mcmillan AM, Roblee KJ. Population Status of Hellbender Salamanders (*Cryptobranchus alleganiensis*) in the Allegheny River Drainage of New York State. J Herpetol. 2009;43(4):579–88.

[35] Mayasich J, Phillips C. Eastern Hellbender Status Assessment Report. US Fish Wildl Serv. 2003;

[36] Burgmeier NG, Sutton TM, Williams RN. Spatial Ecology of the Eastern Hellbender (*Cryptobranchus alleganiensis alleganiensis*) in Indiana. Herpetologica. 2011;67(2):135–45. 10.7589/0090-3558-47.4.836 22102654

[37] Freake MJ, O’Neill E, Unger S, Spear S, Routman E. Conservation genetics of eastern hellbenders *Cryptobranchus alleganiensis alleganiensis* in the Tennessee Valley. Conserv Genet [Internet]. 2018;19(3):571–85. 10.1007/s10592-017-1033-8

[38] Abell RA, Olson DM, Dinerstein E, Hurley PT, Eichbaum W, Diggs JT, et al. Freshwater Ecoregions of North America: a conservation assessment. Washington, D.C.: Island Press; 2000.

[39] Freake MJ, De Perno CS. Importance of demographic surveys and public lands for the conservation of eastern hellbenders *Cryptobranchus alleganiensis* alleganiensis in southeast USA. PLoS One. 2017;12(6):1–16. 10.1371/journal.pone.0179153 PMC5464636 28594881

[40] Bodinof CM, Briggler JT, Junge RE, Mong T, Beringer J, Wanner MD, et al. Survival and Body Condition of Captive-Reared Juvenile Ozark Hellbenders (*Cryptobranchus alleganiensis bishopi*) Following Translocation to the Wild. Copeia [Internet]. 2012;2012(1):150–9. http://www.bioone.org/doi/abs/10.1643/CH-11-024

[41] Gates JE, Stouffer RH, Stauffer JR Jr., Hocutt CH. Dispersal Patterns of Translocated *Cryptobranchus alleganiensis* in a Maryland Stream. J Herpetol. 1985;19(3):436–8.

[42] Bodinof CM, Briggler JT, Junge RE, Beringer J, Wanner MD, Schuette CD, et al. Habitat attributes associated with short-term settlement of Ozark hellbender (*Cryptobranchus alleganiensis bishopi*) salamanders following translocation to the wild. Freshw Biol. 2012;57(1):178–92.

[43] Strahler AN. Quantitative analysis of watershed geomorphology. Trans Am Geophys Union. 1957;38(6):913–20.

[44] Da Silva Neto JG, Sutton WB, Spear SF, Freake MJ, Kéry M, Schmidt BR. Integrating species distribution and occupancy modeling to study hellbender (*Cryptobranchus alleganiensis*) occurrence based on eDNA surveys. Biol Conserv [Internet]. 2020;251(October):108787. 10.1016/j.biocon.2020.108787

[45] Nickerson MA, Krysko KL. Surveying for hellbender salamanders, *Cryptobranchus alleganiensis* (Daudin): a review and critique. Appl Herpetol [Internet]. 2003;1(1):37–44. http://booksandjournals.brillonline.com/content/10.1163/157075403766451216

[46] Nolan E, Nissen B, Sutton W, Freake M, Hardman R. Translocation does not influence amphibian chytrid fungus prevalence among translocated wild eastern hellbenders *Cryptobranchus alleganiensis*. Dis Aquat Organ [Internet]. 2021;145:145–57. 10.3354/dao03607 34196284

[47] Hime PM, Briggler JT, Reece JS, Weisrock DW. Genomic Data Reveal Conserved Female Heterogamety in Giant Salamanders with Gigantic Nuclear Genomes. G3 (Bethesda). 2019;9(10):3467–76. doi: 10.1534/g3.119.400556 31439718 PMC6778777

[48] Connock JR, Case BF, Button ST, Groffen J, Galligan TM, Hopkins WA. Factors influencing in-situ detection of PIT-tagged Hellbenders (*Cryptobranchus alleganiensis*) occupying artificial shelters using a submersible antenna. Herpetol Conserv Biol [Internet]. 2019;14(2):429–37.

[49] Button ST, Bodinof Jachowski CM, Case BF, Groffen J, Hopkins WA. The Influence of Multiscale Habitat Variables and Population Density on Artificial Shelter Use by Hellbenders (*Cryptobranchus alleganiensis*). Herpetologica. 2020;76(4):355–65.

[50] Hansen EA, Closs GP. Diel activity and home range size in relation to food supply in a drift-feeding stream fish. Behav Ecol. 2005;16(3):640–8.

[51] Humphries WJ. Diurnal Seasonal Activity of *Cryptobranchus alleganiensis* (Hellbender) in North Carolina. Southeast Nat. 2007;6(1):135–40.

[52] Peterson CL, Reed JW, Wilkinson RF. Seasonal Food Habits of *Cryptobranchus alleganiensis* (Caudata: Cryptobranchidae). Southwest Nat. 1989;34(3):438–41.

[53] Augustine L, Terrell KA, Nissen B, Maslanka M. Nutritional Analysis of Diet Items Available to Captive and Free-ranging Hellbenders (*Cryptobranchus alleganiensis*). Herpetol Rev. 2016;47(1):63–9.

[54] Mather ME, Stein RA. Direct and indirect effects of fish predation on the replacement of a native crayfish by an invading congener. Can J Fish Aquat Sci. 1993;50(6):1279–88.

[55] Daugherty DJ, Sutton TM. Seasonal Movement Patterns, Habitat Use, and Home Range of Flathead Catfish in the Lower St. Joseph River, Michigan. North Am J Fish Manag. 2005;25(1):256–69.

[56] Row JR, Blouin-Demers G. Kernels are not Accurate Estimators of Home-Range Size For Herpetofauna. Copeia. 2006;(4):797–802.

[57] Tarjan LM, Tinker MT. Permissible home range estimation (PHRE) in restricted habitats: A new algorithm and an evaluation for sea otters. PLoS One. 2016;11(3). doi: 10.1371/journal.pone.0150547 27003710 PMC4803229

[58] Nissen BD. Assessing the Efficacy of Translocation as a Conservation Strategy for Wild Eastern Hellbenders (*Cryptobranchus alleganiensis alleganiensis*) in Tennessee [Internet]. Tennessee State University; 2020. https://www.proquest.com/openview/5b08209687cd9e221ae186563230c9bb/1?pq-origsite=gscholar&cbl=18750&diss=y

[59] Mohr CO. Table of Equivalent Populations of North American Small Mammals. Am Midl Nat. 1947;37(1):223.

[60] Ross JP, Bluett RD, Dreslik MJ. Movement and home range of the smooth softshell turtle (*Apalone mutica*): Spatial ecology of a river specialist. Diversity. 2019;11(8).

[61] McCullagh P, Nelder JA. Binary data. In: Generalized linear models. Springer US; 1989. p. 98–148.

[62] Burnham KP, Anderson DR. Model Selection and Multimodel Inference. Second edi. Springer US; 2002.

[63] Mazerolle MJ. AICcmodavg. R Package 281; 2019.

[64] Boyce MS, Vernier PR, Nielsen SE, Schmiegelow FKA. Evaluating resource selection functions. Ecol Modell. 2002;157(2–3):281–300.

[65] Pinjuv G, Mason EG, Watt M. Quantitative validation and comparison of a range of forest growth model types. For Ecol Manage. 2006;236(1):37–46.

[66] Hecht K, Nickerson MA. *Cryptobranchus alleganiensis* (hellbender) predation. Herpetol Rev. 2014;(September 2014).

[67] Ball BS. Habitat use and movements of Eastern Hellbenders, *Cryptobranchus alleganiensis alleganiensis*: a radiotelemetric study. Vol. MS thesis, Department of Biology. Appalachian State University; 2001.

[68] Nickerson MA, Mays CE. A Study of the Ozark Hellbender *Cryptobranchus alleganiensis bishopi*. Ecology. 1973;54(5):1164–5.

[69] Nyström P, Stenroth P, Holmqvist N, Berglund O, Larsson P, Granéli W. Crayfish in lakes and streams: Individual and population responses to predation, productivity and substratum availability. Freshw Biol. 2006;51(11):2096–113.

[70] Coatney Jr. CE. Home range and nocturnal activity of the Ozark hellbender [Internet]. Southwest Missouri State University; 1982. https://ag.purdue.edu/fnr/discover/HerpetologyLab/Documents/Coatney_HomeRange.pdf

[71] Humphries WJ, Pauley TK. Seasonal Changes in Nocturnal Activity of the Hellbender, *Cryptobranchus alleganiensis*, in West Virginia. J Herpetol. 2000;34(4):604–7.

[72] Hecht K, Nickerson MA, Freake M, Colclough P, Stofer K. Body Condition in Three Hellbender Populations. BioRxiv. 2019;1–21.

[73] Smith BG. The Life History and Habits of *Cryptobranchus allegheniensis*. Contrib from Zool Lab Univ Michigan. 1907;109(5):5–39.

[74] Tonitto C, Riha SJ. Planning and implementing small dam removals: lessons learned from dam removals across the eastern United States. Sustain Water Resour Manag. 2016;2(4):489–507.

[75] Haag WR, Williams JD. Biodiversity on the brink: An assessment of conservation strategies for North American freshwater mussels. Hydrobiologia. 2014;735(1):45–60.

[76] Monk CT, Chéret B, Czapla P, Hühn D, Klefoth T, Eschbach E, et al. Behavioural and fitness effects of translocation to a novel environment: Whole-lake experiments in two aquatic top predators. J Anim Ecol. 2020;(June 2019):1–20. doi: 10.1111/1365-2656.13298 32654123

[77] Pugh MW, Franklin T, Siefferman L, Gangloff MM. A protocol for quantifying hellbender abundance and in-stream habitat. Herpetol Conserv Biol. 2018;13(3):598–608.

