# Supplementary figures and images for: Evaluating translocation success of wild eastern hellbenders (Cryptobranchus alleganiensis alleganiensis) in Blue Ridge Ecoregion streams using pre- and post-translocation home range sizes and movement metrics
Source: PLoS One. 2023 Apr 20;18(4):e0283377. doi: 10.1371/journal.pone.0283377 (PMC10118149; doi:10.1371/journal.pone.0283377)

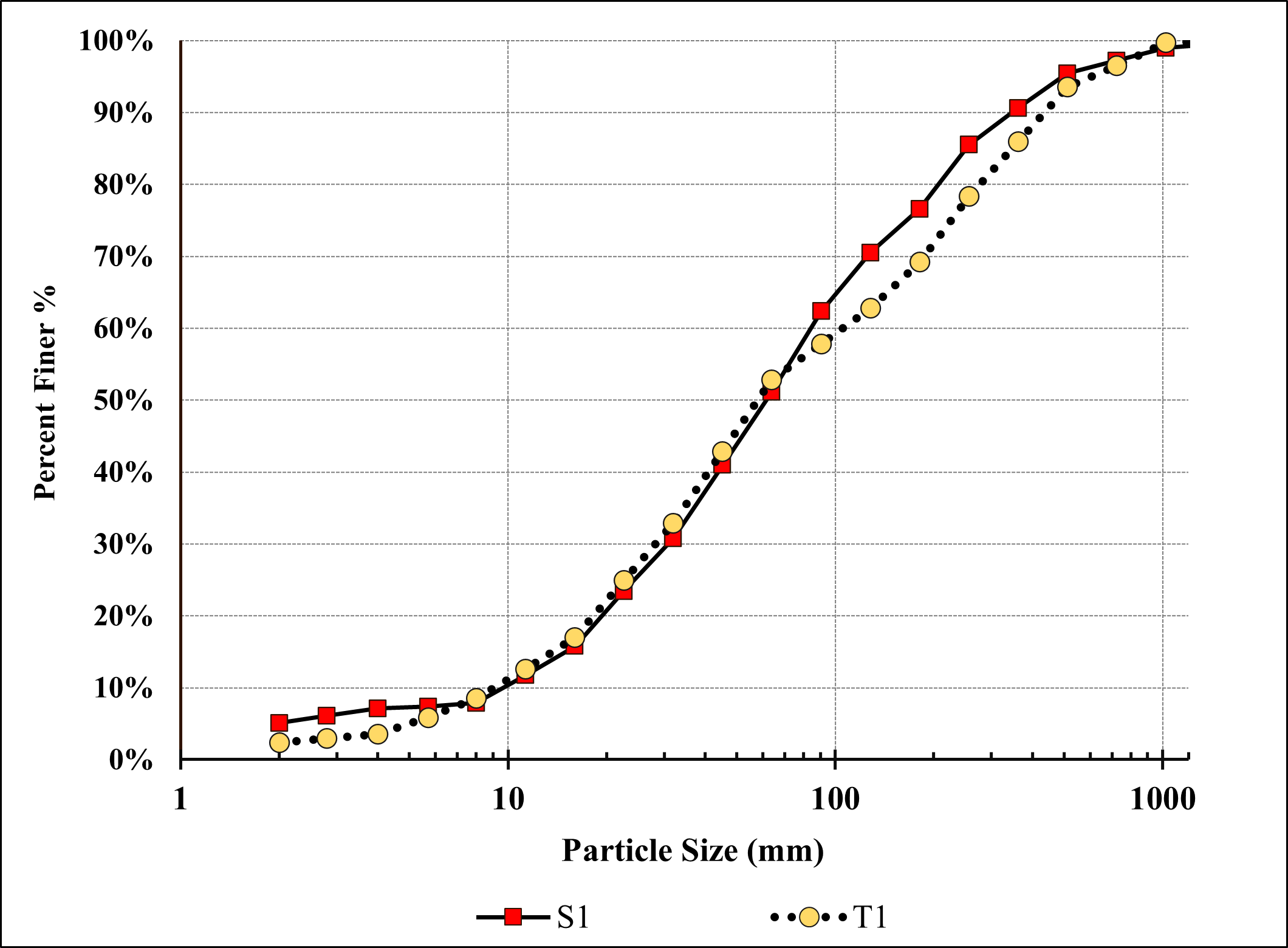

Supplement: S1 Fig — Comparison of the distributions of substrate sizes for each stream in the study, comparing source and translocation sites by watershed. Pebble counts were conducted at all streams following methods standardized for quantifying hellbender habitat [77]. (TIF) [file pone.0283377.s001.tif]

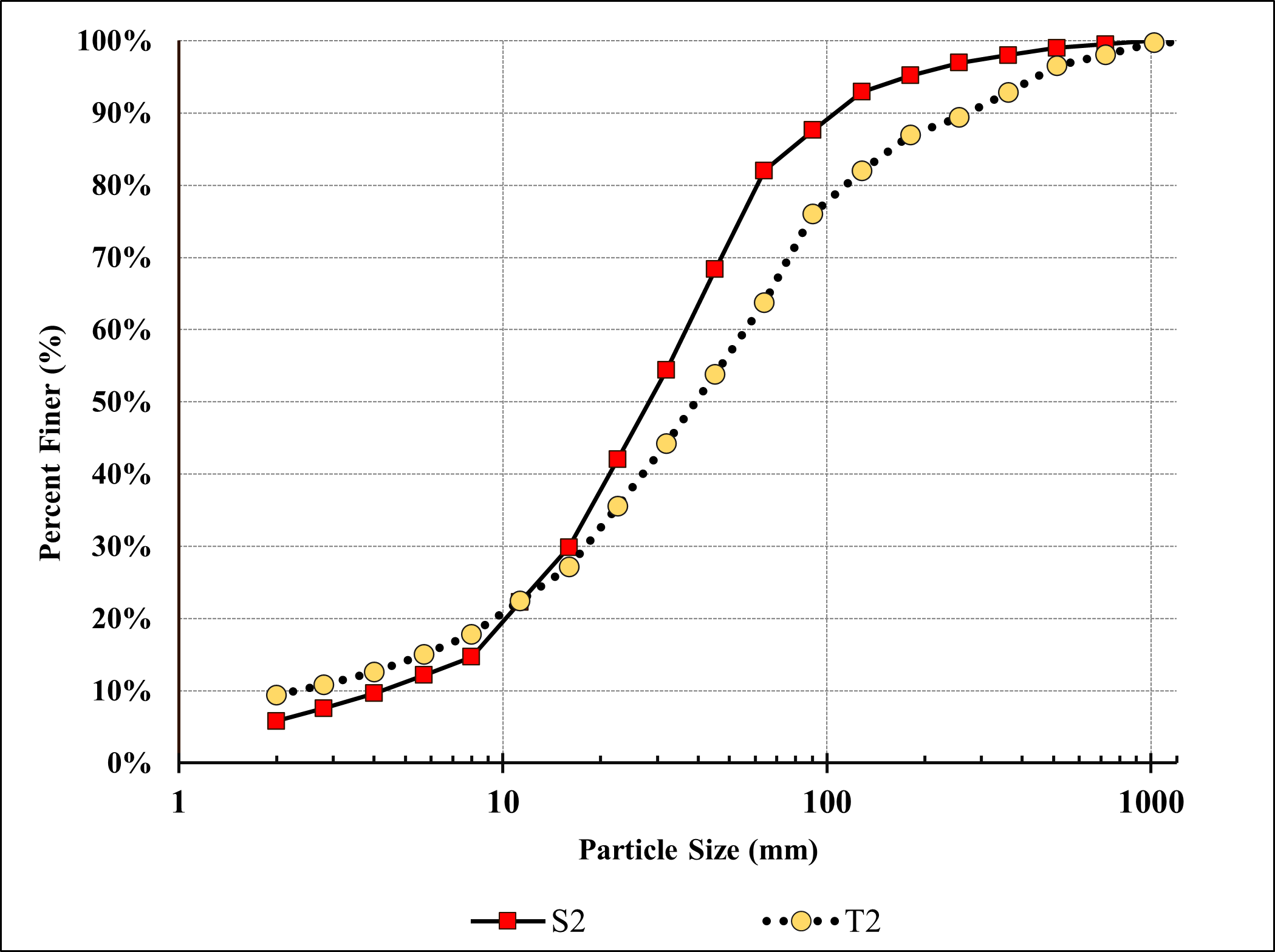

Supplement: S2 Fig — Comparison of the distributions of substrate sizes for each stream in the study, comparing source and translocation sites by watershed. Pebble counts were conducted at all streams following methods standardized for quantifying hellbender habitat [77]. (TIF) [file pone.0283377.s002.tif]

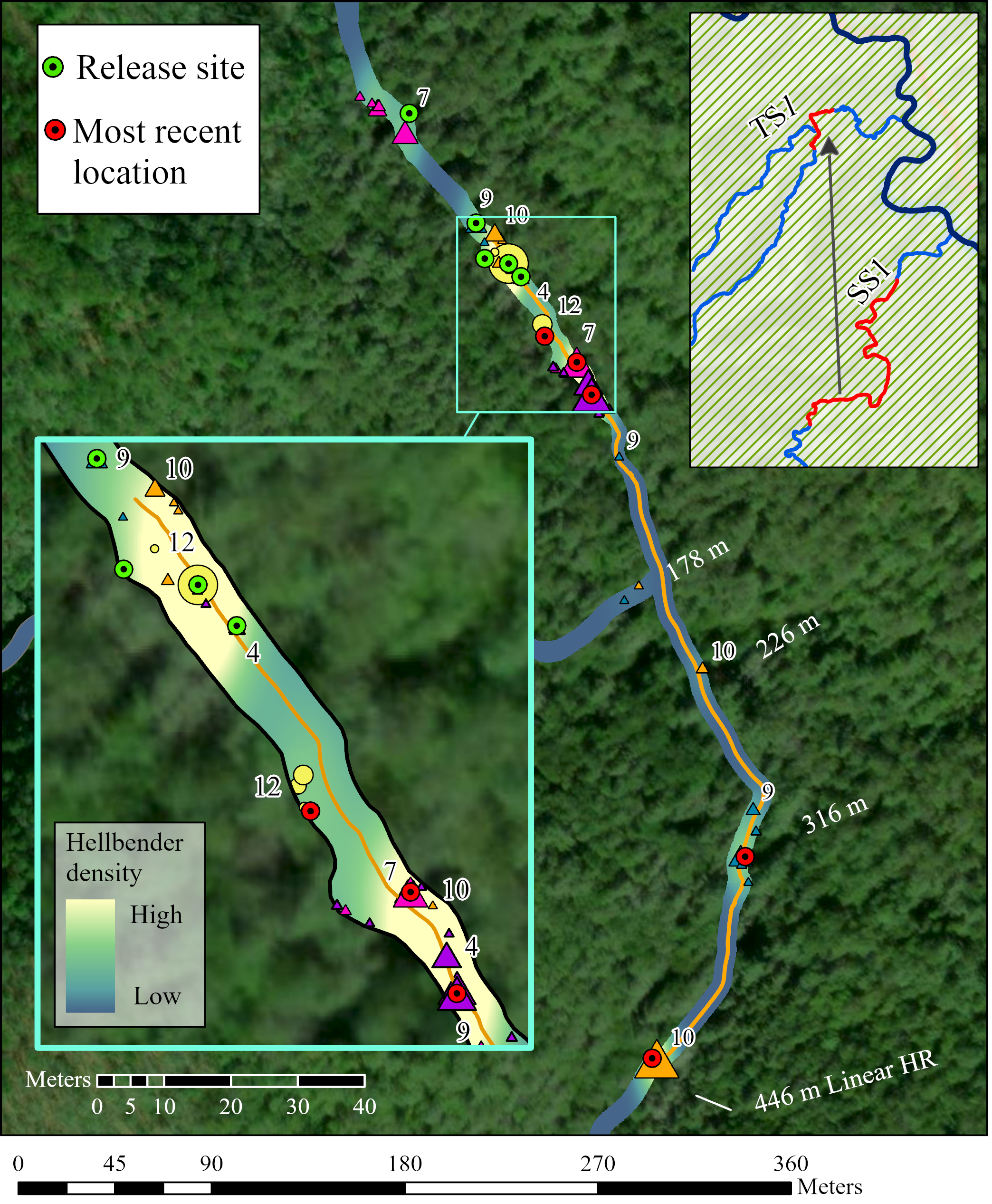

Supplement: S3 Fig — Map displaying individual hellbender locations and movement directions at translocation site 1 from 2019–2020. Hellbender locations are given in circles (females) or triangles (males), and each color represents locations of a different hellbender. Colors are labeled by individual ID (i.e., 4, 7, 9, 10 or 12). Sizes of the location markers are proportional to the number of times an individual was observed at that location, with more locations having larger markers. Permissible home range estimates are used to showcase where hellbenders were most frequently observed (inset map) during the year-long post-translocation season (2019–2020). The orange line depicts the linear home range (LHR) of individual #10 –with labels indicating distances traveled from the release site. Spatial imagery and files sourced from USGS National Map Viewer. (TIF) [file pone.0283377.s003.tif]

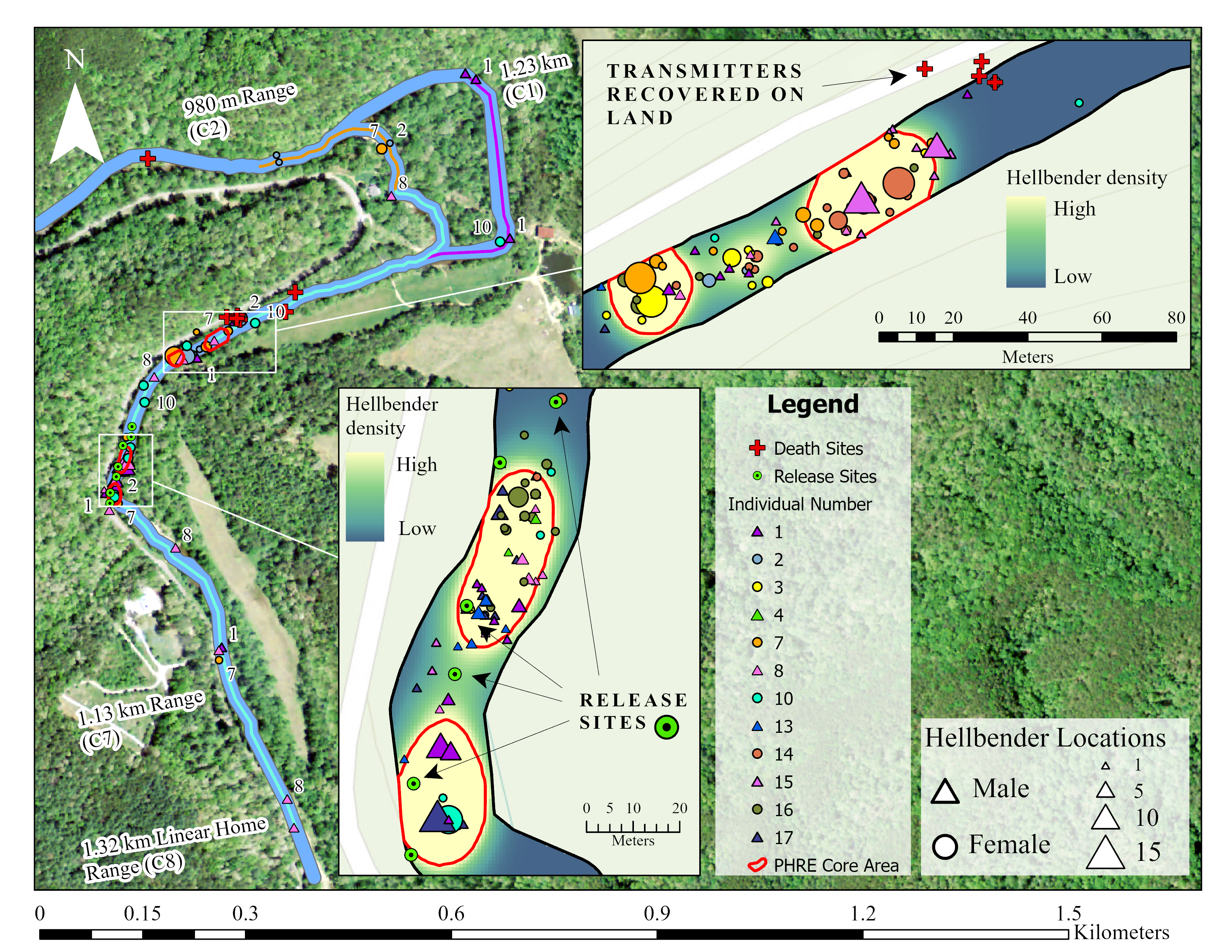

Supplement: S4 Fig — Map displaying individual hellbender locations and movement directions at translocation site 2. Hellbender locations are given in circles (females) or triangles (males), and each color represents locations of a different hellbender. Numbered labels along the river also denote individual IDs. Sizes of the location markers are proportional to the number of times an individual was observed at that location, with more locations having larger markers. Permissible home range estimates are used to showcase where hellbenders were most frequently observed (red circles; inset maps) over the year-long 2019–2020 sampling season. The colored lines depict the linear home range (LHR) of individuals–with labels indicating individual ID and total distances traveled between the most extreme points for that individual. Spatial imagery and files sourced from USGS National Map Viewer. (TIF) [file pone.0283377.s004.tif]

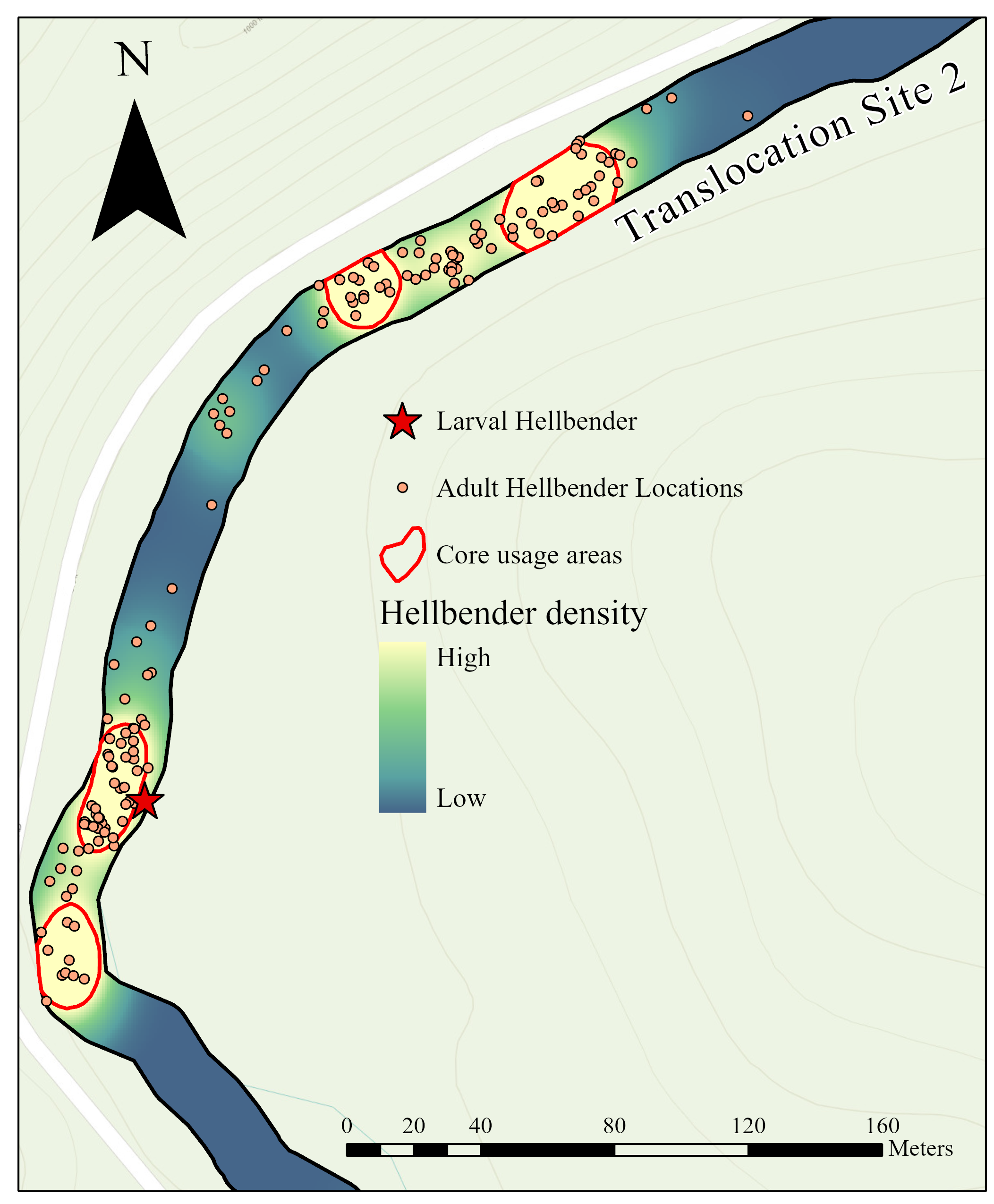

Supplement: S5 Fig — A simple map displaying where a larval hellbender was found at T2 in relation to the areas used most frequently by the translocated hellbenders at that site. (TIF) [file pone.0283377.s005.tif]
