# Supplementary material for: Evaluating translocation success of wild eastern hellbenders (Cryptobranchus alleganiensis alleganiensis) in Blue Ridge Ecoregion streams using pre- and post-translocation home range sizes and movement metrics
Source: PLoS One. 2023 Apr 20;18(4):e0283377. doi: 10.1371/journal.pone.0283377 (PMC10118149; doi:10.1371/journal.pone.0283377)
Supplement: S1 Table — A priori models representing hypotheses, model structures, and predicted effects for factors associated with the spatial ecology (i.e. movement behaviors and home range sizes) of Eastern Hellbenders. For analyses 3 and 4 (translocated individuals only), models that included translocation status as a covariate (“Trans”) were tested by replacing “Trans” with the covariate cohort. In cases of interactive terms, the interaction with “Trans” was dropped and Cohort was used alone. Random effects of individual were not included in analysis 3 and 4. *—model tested only during analysis of translocated individuals. (DOCX) [file pone.0283377.s006.docx]

## Table S1. Spatial Ecology Models

*A priori* models representing hypotheses, model structures, and predicted effects for factors associated with the spatial ecology (i.e. movement behaviors and home range sizes) of Eastern Hellbenders. For analyses 3 and 4 (translocated individuals only), models that included translocation status as a covariate (“Trans”) were tested by replacing “Trans” with the covariate cohort. In cases of interactive terms, the interaction with “Trans” was dropped and Cohort was used alone. Random effects of individual were not included in analysis 3 and 4.

* - model tested only during analysis of translocated individuals.

| Model name | Hypothesis | Covariates/Model Structure | Predicted effects |
| --- | --- | --- | --- |
| Global | Hellbender spatial ecology depends on all measured covariates | Sex + Cohort*Trans + R_SIZE_ + R_DENS_ + Mass + (1\|ID) | β_Sex:Male_ > 0; β_R-Size_ > 0; β_R-Dens_ < 0; β_Mass_ < 0; β_Cohort[2]_ < 0; β_Trans[1]_ > 0;  β_Cohort[2]*Trans[1]_ < 0 |
| Nested Global | Hellbender spatial ecology depends on all measured covariates, and the effect of habitat variables is dependent upon the site | Sex + Trans/R_SIZE_ + Trans/R_DENS_ + Mass + (1\|ID) | β_Sex:Male_ > 0; β_Trans/R-Size_ > 0;  β _Trans/R-Dens_ < 0; β_Mass_ < 0 |
| Environment + Status | Hellbender spatial ecology depends on their environment and translocation status. | R_SIZE_ + R_DENS_ + Cohort*Trans + (1\|ID) | β_R-Size_ > 0; β_R-Dens_ < 0; β_Cohort[2]_ < 0; β_Trans[1]_ > 0;  β_Cohort[2]*Trans[1]_ < 0 |
| Nested Environment | Hellbender spatial ecology depends on substrate characteristics, which are dependent upon translocation status. | Trans/R_SIZE_ + Trans/R_DENS_ | β _Trans/R-Size_ > 0;  β _Trans/R-Dens_ < 0 |
| Nested Rock Size | Hellbender spatial ecology depends only on the sizes of cover rocks, which is dependent upon translocation status | Trans/R_SIZE_ | β_Site/R-Size_ > 0 |
| Nested Rock Density | Hellbender spatial ecology depends only on the density of cover rocks, which is dependent upon translocation status | Trans/R_DENS_ | β _Site/R-Dens_ < 0 |
| Individual | Hellbender spatial ecology depends only on individual characteristics of sex and size | Mass + Sex | β_Sex:Male_ > 0; β_Mass_ < 0 |
| Status | Hellbender spatial ecology depends only on translocation status and unmeasured site characteristics | Cohort*Trans | β_Cohort[2]_ < 0; β_Trans[1]_ > 0;  β_Cohort[2]*Trans[1]_ < 0 |
| Pre-Trans. LHR* | Hellbender spatial ecology of translocated individuals depends only on the size of the individual’s LHR prior to translocation. | LHR_PRE_ | Β_LHR-Pre_ < 0 |
| Pre-Trans. Sedent* | Hellbender spatial ecology of translocated individuals depends only on the individual’s sedentariness prior to translocation. | SEDENT_PRE_ | Β_SEDENT-Pre_ < 0 |
| Pre-Trans. Sedent + LHR* | Hellbender spatial ecology of translocated individuals depends on the individual’s sedentariness and LHR size prior to translocation. | SEDENT_PRE_ + LHR_PRE_ | Β_LHR-Pre_ < 0; Β_SEDENT-Pre_ < 0 |
| Substrate + Pre-Trans HR* | Hellbender spatial ecology of translocated individuals depends on substrate characteristics and individual’s spatial ecology prior to translocation | R_SIZE_ + R_DENS_ + SEDENT_PRE_ + LHR_PRE_ | β_R-Size_ > 0; β_R-Dens_ < 0; Β_LHR-Pre_ < 0; Β_SEDENT-Pre_ < 0 |
| Substrate + Pre-Trans Sedent* | Hellbender spatial ecology of translocated individuals depends on substrate characteristics and individual’s sedentariness prior to translocation | R_SIZE_ + R_DENS_ + SEDENT_Pre_ | β_R-Size_ > 0; β_R-Dens_ < 0; Β_SEDENT-Pre_ < 0 |
| Substrate + Pre-Trans LHR* | Hellbender spatial ecology of translocated individuals depends on substrate characteristics and individual’s LHR size prior to translocation | R_SIZE_ + R_DENS_ + LHR_PRE_ | β_R-Size_ > 0; β_R-Dens_ < 0; Β_LHR-Pre_ < 0 |
| Null | None of the variables explain the variability in hellbender spatial ecology | Intercept only |  |
