# Supplementary material for: Evaluating translocation success of wild eastern hellbenders (Cryptobranchus alleganiensis alleganiensis) in Blue Ridge Ecoregion streams using pre- and post-translocation home range sizes and movement metrics
Source: PLoS One. 2023 Apr 20;18(4):e0283377. doi: 10.1371/journal.pone.0283377 (PMC10118149; doi:10.1371/journal.pone.0283377)
Supplement: S2 Table — Summary statistics of movement metrics and habitat data by individual hellbender for S1-T1 cohort (S2 Table) and S2-T2 cohort (S3 Table). Post-translocation metrics (colored) are presented for all individuals that were translocated. Where applicable, averages are given with standard errors. Trans. = Translocation. Loc. = Locations. Dist. = Distance. Sedent. = Sedentariness. Dens. = Density. ♀ = Female; ♂ = Male. * = less than 15 locations. (DOCX) [file pone.0283377.s007.docx]

## Table S2 & S3. Individual Movement Metrics and Habitat Data.

Summary statistics of movement metrics and habitat data by individual hellbender for S1-T1 cohort (Table S2) and S2-T2 cohort (Table S3). Post-translocation metrics (colored) are presented for all individuals that were translocated. Where applicable, averages are given with standard errors. Trans. = Translocation. Loc. = Locations. Dist. = Distance. Sedent. = Sedentariness. Dens. = Density. ♀ = Female; ♂ = Male. * = less than 15 locations.

| **Cohort – Source Site 1 to Translocation Site 1** | | | | | | | | | |
| --- | --- | --- | --- | --- | --- | --- | --- | --- | --- |
| **ID** | **Trans. Status** | **Loc.** | **Moves** | **Avg. Movement Size (m)** | **Avg. Daily Movement (m)** | **Total Dist. (m)** | **Sedent.** | **Avg. Cover Rock Size (cm)** | **Avg. Dens. of Cover Rocks** |
| ♂ **4** | Pre | 45 | 22 | 9.2 ± 1.2 | 4.0 ± 1.0 | 202 | 0.51 | 70.8 ± 6.5 | 8.7 ± 0.7 |
|  | Post | 58 | 16 | 13.8 ± 3.5 | 3.2 ± 0.9 | 207 | 0.72 | 68.4 ± 3.1 | 5.9 ± 0.3 |
| ♀ **5** | Pre | 45 | 18 | 12.3 ± 2.3 | 2.2 ± 0.7 | 222 | 0.60 | 47.8 ± 2.4 | 7.3 ± 0.8 |
| ♀ **6** | Pre | 58 | 28 | 9.0 ± 0.9 | 2.8 ± 0.4 | 244 | 0.52 | 73.1 ± 3.1 | 7.2 ± 0.4 |
| ♂ **7** | Pre | 42 | 30 | 11.1 ± 2.8 | 3.0 ± 0.7 | 311 | 0.29 | 47.9 ± 2.6 | 9.1 ± 0.5 |
|  | Post | 59 | 10 | 26.9 ± 13.8 | 11.7 ± 4.4 | 270 | 0.83 | 79.2 ± 2.5 | 6.1 ± 0.6 |
| ♂ **8** | Pre | 85 | 51 | 50.0 ± 12.8 | 11.0 ± 2.6 | 2600 | 0.40 | 69.1 ± 6.2 | 6.7 ± 0.3 |
| ♂ **9** | Pre | 55 | 27 | 25.5 ± 11.5 | 4.7 ± 1.2 | 715 | 0.51 | 55.1 ± 5.0 | 7.7 ± 0.4 |
|  | Post | 43 | 12 | 32.0 ± 10.95 | 10.1 ± 2.9 | 384 | 0.72 | 62.7 ± 2.0 | 5.3 ± 0.5 |
| ♂ **10** | Pre | 41 | 12 | 11.66 ± 1.7 | 2.0 ± 0.6 | 140 | 0.71 | 78.9 ± 2.9 | 6.5 ± 0.3 |
|  | Post | 57 | 13 | 45.77 ± 18.9 | 13.3 ± 5.0 | 458 | 0.77 | 55.9 ± 2.0 | 6.1 ± 0.5 |
| ♂ **11** | Pre | 45 | 24 | 17.17 ± 4.25 | 4.8 ± 1.0 | 498 | 0.47 | 106.6 ± 5.1 | 6.9 ± 0.3 |
| ♀ **12** | Pre | 40 | 21 | 10.5 ± 1.0 | 3.7 ± 1.1 | 231 | 0.47 | 55.1 ± 1.9 | 7.2 ± 0.3 |
|  | Post | 59 | 7 | 9.37 ± 6.34 | 2.6 ± 1.4 | 56 | 0.88 | 72.3 ± 1.7 | 5.0 ± 0.2 |
| ♀ **13** | Pre | 64 | 18 | 20.4 ± 5.0 | 5.4 ± 1.3 | 387 | 0.72 | 238.3 ± 19.3 | 5.6 ± 0.3 |
