# Supplementary material for: Evaluating translocation success of wild eastern hellbenders (Cryptobranchus alleganiensis alleganiensis) in Blue Ridge Ecoregion streams using pre- and post-translocation home range sizes and movement metrics
Source: PLoS One. 2023 Apr 20;18(4):e0283377. doi: 10.1371/journal.pone.0283377 (PMC10118149; doi:10.1371/journal.pone.0283377)
Supplement: S3 Table — Summary statistics of movement metrics and habitat data by individual hellbender for S1-T1 cohort (S2 Table) and S2-T2 cohort (S3 Table). Post-translocation metrics (colored) are presented for all individuals that were translocated. Where applicable, averages are given with standard errors. Trans. = Translocation. Loc. = Locations. Dist. = Distance. Sedent. = Sedentariness. Dens. = Density. ♀ = Female; ♂ = Male. * = less than 15 locations. (DOCX) [file pone.0283377.s008.docx]

## Table S2 & S3. Individual Movement Metrics and Habitat Data.

Summary statistics of movement metrics and habitat data by individual hellbender for S1-T1 cohort (Table S2) and S2-T2 cohort (Table S3). Post-translocation metrics (colored) are presented for all individuals that were translocated. Where applicable, averages are given with standard errors. Trans. = Translocation. Loc. = Locations. Dist. = Distance. Sedent. = Sedentariness. Dens. = Density. ♀ = Female; ♂ = Male. * = less than 15 locations.

| **Cohort – Source Site 2 to Translocation Site 2** | | | | | | | | | |
| --- | --- | --- | --- | --- | --- | --- | --- | --- | --- |
| **ID** | **Trans. Status** | **Loc.** | **Moves** | **Avg. Movement Size (m)** | **Avg. Daily Movement (m)** | **Total Dist. (m)** | **Sedent.** | **Avg. Cover Rock Size (cm)** | **Avg. Dens. of Cover Rocks** |
| ♂ **1** | Pre | 25 | 15 | 9.0 ± 2.3 | 2.7 ± 1.8 | 125 | 0.40 | 44.5 ± 3.8 | 3.7 ± 0.5 |
|  | Post | 47 | 26 | 152 ± 36.5 | 50 ± 15.5 | 3800 | 0.45 | 54.1 ± 3.7 | 8.0 ± 0.6 |
| ♀ **2** | Pre | 19 | 5 | 3.1 ± 1.4 | 0.4 ± 0.3 | 13 | 0.74 | 52.5 ± 3.6 | 3.4 ± 0.5 |
|  | Post | 15 | 10 | 176.3 ± 42.9 | 83.0 ± 25.3 | 1587 | 0.33 | 44.3 ± 2.2 | 12.7 ± 2.2 |
| ♀ **3** | Pre | 19 | 10 | 31.6 ± 15.7 | 4.3 ± 2.4 | 284 | 0.47 | 58.9 ± 2.4 | 4.5 ± 0.5 |
|  | Post | 44 | 17 | 47.1 ± 11.2 | 19.4 ± 4.9 | 847 | 0.61 | 51.0 ± 4.6 | 4.9 ± 0.5 |
| ♂ **4** | Pre | 25 | 8 | 24.9 ± 2.6 | 4.5 ± 2.8 | 174 | 0.68 | 63.2 ± 1.7 | 0.5 ± 0.1 |
|  | Post* | 9 | 5 | 49.3 ± 20.9 | 38.12 ± 23.1 | 197 | 0.44 | 63.7 ± 5.5 | 5.7 ± 0.8 |
| ♂ **5** | Pre | 15 | 7 | 32.1 ± 10.8 | 8.1 ± 3.9 | 193 | 0.53 | 51.0 ± 4.3 | 2.4 ± 0.5 |
| ♀ **6** | Pre* | 10 | 6 | 12.5 ± 3.0 | 5.0 ± 3.4 | 63 | 0.40 | 59.6 ± 3.0 | 1.0 ± 0.5 |
| ♀ **7** | Pre | 26 | 15 | 15.0 ± 2.6 | 2.6 ± 0.5 | 209 | 0.42 | 50.6 ± 1.9 | 2.3 ± 0.3 |
|  | Post | 48 | 18 | 179 ± 49 | 74 ± 18.9 | 2863 | 0.63 | 65.8 ± 5.9 | 4.9 ± 0.4 |
| ♂ **8** | Pre | 26 | 11 | 13.6 ± 3.2 | 2.6 ± 1.7 | 150 | 0.58 | 69.9 ± 7.3 | 2.2 ± 0.5 |
|  | Post | 23 | 18 | 273.4 ± 55.6 | 90.2 ± 22.3 | 4648 | 0.22 | 48.6 ± 6.2 | 8.0 ± 1.1 |
| ♀ **9** | Pre | 15 | 8 | 15.2 ± 5.17 | 2.6 ± 0.9 | 106 | 0.47 | 69.8 ± 1.0 | 0.8 ± 0.2 |
| ♀ **10** | Pre | 24 | 4 | 3.6 ± 0.8 | 0.5 ± 0.1 | 11 | 0.83 | 53.9 ± 1.8 | 3.9 ± 0.3 |
|  | Post | 24 | 11 | 191.6 ± 60.1 | 107.2 ± 53.3 | 1916 | 0.54 | 59.5 ± 5.4 | 5.6 ± 0.8 |
| ♂ **11** | Pre | 33 | 8 | 7.2 ± 2.1 | 2.7 ± 1.8 | 65 | 0.76 | 73.5 ± 6.0 | 1.5 ± 0.2 |
| ♀ **12** | Pre* | 13 | 3 | 14.3 ± 0.8 | 1.6 ± 0.6 | 29 | 0.77 | 70.6 ± 5.5 | 1.4 ± 0.4 |
| ♂ **13** | Pre | 23 | 4 | 11.1 ± 7.7 | 1.2 ± 0.4 | 33 | 0.83 | 73.0 ± 1.9 | 1.8 ± 0.1 |
|  | Post | 22 | 14 | 91.2 ± 33.8 | 42.1 ± 17.7 | 1186 | 0.36 | 47.5 ± 2.6 | 9.0 ± 1.0 |
| ♀ **14** | Pre* | 14 | 10 | 11.6 ± 1.8 | 1.5 ± 0.4 | 104 | 0.29 | 48.3 ± 2.7 | 7.9 ± 1.0 |
|  | Post | 64 | 26 | 29.1 ± 8.0 | 12.3 ± 3.3 | 757 | 0.59 | 62.6 ± 3.3 | 6.2 ± 0.4 |
| ♂ **15** | Pre | 15 | 7 | 13.2 ± 2.5 | 1.9 ± 0.5 | 79 | 0.53 | 64.0 ± 4.5 | 5.9 ± 0.5 |
|  | Post | 64 | 18 | 36.6 ± 16.0 | 12.3 ± 5.6 | 659 | 0.72 | 90.3 ± 2.3 | 4.8 ± 0.5 |
| ♀ **16** | Pre | 15 | 8 | 18.34 ± 8.4 | 2.9 ± 1.5 | 128 | 0.47 | 59.7 ± 7.7 | 13.5 ± 1.3 |
|  | Post | 46 | 29 | 66.17 ± 13.7 | 23.9 ± 5.2 | 1853 | 0.37 | 59.1 ± 3.4 | 7.8 ± 0.8 |
| ♂ **17** | Pre* | 12 | 5 | 10.39 ± 0.3 | 3.0 ± 2.3 | 42 | 0.58 | 57.9 ± 7.9 | 4.5 ± 0.5 |
|  | Post | 40 | 21 | 36.8 ± 11.7 | 16.9 ± 4.7 | 736 | 0.48 | 59.1 ± 2.4 | 11.1 ± 0.8 |
