# Supplementary material for: Evaluating translocation success of wild eastern hellbenders (Cryptobranchus alleganiensis alleganiensis) in Blue Ridge Ecoregion streams using pre- and post-translocation home range sizes and movement metrics
Source: PLoS One. 2023 Apr 20;18(4):e0283377. doi: 10.1371/journal.pone.0283377 (PMC10118149; doi:10.1371/journal.pone.0283377)
Supplement: S4 Table — Summary statistics of home range sizes by individual hellbender for S1-T1 cohort. Pre- and post-translocation metrics are presented for all individuals that were translocated; post-translocation rows are colored. Kernel density estimates (KDEs) and permissible home range estimates (PHREs) were only calculated for individuals with more than 20 locations at a site. Trans. = Translocation. LHR = Linear home range. MCP = Minimum convex polygon home range. ♀ = Female; ♂ = Male. (DOCX) [file pone.0283377.s009.docx]

## Table S4. Individual Home Range Sizes.

Summary statistics of home range sizes by individual hellbender for S1-T1 cohort. Pre- and post-translocation metrics are presented for all individuals that were translocated; post-translocation rows are colored. Kernel Density estimates (KDEs) and Permissible Home Range estimates (PHREs) were only calculated for individuals with more than 20 locations at a site. Trans. = Translocation. LHR = Linear Home Range. MCP = Minimum Convex Polygon home range. ♀ = Female; ♂ = Male.

| **Cohort – Source Site 1 to Translocation Site 1** | | | | | | | | |
| --- | --- | --- | --- | --- | --- | --- | --- | --- |
| **Animal ID** | **Trans. Status** | **Locations** | **LHR (m)** | **MCP (m^2^)** | **50%**  **KDE (m^2^)** | **95% KDE (m^2^)** | **50%**  **PHRE (m^2^)** | **95%**  **PHRE (m^2^)** |
| ♂ **4** | Pre | 45 | 31.90 | 249.24 | 59.33 | 271.2 | 27.43 | 160.06 |
|  | Post | 58 | 81.24 | 516.7 | 40.43 | 300.44 | 42.02 | 284.19 |
| ♀ **5** | Pre | 45 | 51.94 | 388.93 | 92.37 | 431.99 | 44.71 | 236.26 |
| ♀ **6** | Pre | 58 | 29.24 | 178.14 | 43.12 | 225.47 | 19.12 | 129.94 |
| ♂ **7** | Pre | 42 | 65.77 | 341 | 83.02 | 289.37 | 50.79 | 244.43 |
|  | Post | 59 | 185.84 | 1296.09 | 124.66 | 959.32 | 302.9 | 813.68 |
| ♂ **8** | Pre | 85 | 2531.95 | 20713.66 | 4468.19 | 22177.9 | 3056.77 | 18000 |
| ♂ **9** | Pre | 55 | 269.23 | 452.68 | 18.65 | 486.39 | 111.78 | 639.07 |
|  | Post | 43 | 350.81 | 1651.44 | 332.05 | 2283.73 | 554.61 | 2353.52 |
| ♂ **10** | Pre | 41 | 24.20 | 152.02 | 7.74 | 91.39 | 3.46 | 43.43 |
|  | Post | 57 | 445.90 | 2025.04 | 264.22 | 1394.38 | 287.47 | 1492.62 |
| ♂ **11** | Pre | 45 | 168.45 | 775.49 | 449.32 | 1339.13 | 293.65 | 1076.47 |
| ♀ **12** | Pre | 40 | 42.69 | 283.65 | 44.46 | 279.94 | 21.57 | 164.9 |
|  | Post | 59 | 46.30 | 107.2 | 5.07 | 45.12 | 17.54 | 95.07 |
| ♀ **13** | Pre | 64 | 100.48 | 773.93 | 109.74 | 673.62 | 66.19 | 462.52 |
