# Supplementary material for: Evaluating translocation success of wild eastern hellbenders (Cryptobranchus alleganiensis alleganiensis) in Blue Ridge Ecoregion streams using pre- and post-translocation home range sizes and movement metrics
Source: PLoS One. 2023 Apr 20;18(4):e0283377. doi: 10.1371/journal.pone.0283377 (PMC10118149; doi:10.1371/journal.pone.0283377)
Supplement: S5 Table — Summary statistics of home range sizes by individual hellbender for S2-T2 cohort. Pre- and post-translocation metrics are presented for all individuals that were translocated; post-translocation rows are colored. Kernel density estimates (KDEs) and permissible home range estimates (PHREs) were only calculated for individuals with more than 20 locations at a site. Trans. = Translocation. LHR = Linear home range. MCP = Minimum convex polygon home range. ♀ = Female; ♂ = Male. (DOCX) [file pone.0283377.s010.docx]

## Table S5. Individual Home Range Sizes.

Summary statistics of home range sizes by individual hellbender for S2-T2 cohort. Pre- and post-translocation metrics are presented for all individuals that were translocated; post-translocation rows are colored. Kernel Density estimates (KDEs) and Permissible Home Range estimates (PHREs) were only calculated for individuals with more than 20 locations at a site. Trans. = Translocation. LHR = Linear Home Range. MCP = Minimum Convex Polygon home range. ♀ = Female; ♂ = Male.

| **Cohort – Source Site 2 to Translocation Site 2** | | | | | | | | |
| --- | --- | --- | --- | --- | --- | --- | --- | --- |
| **Animal ID** | **Trans. Status** | **Locations** | **LHR**  **(m)** | **MCP**  **(m^2^)** | **50%**  **KDE (m^2^)** | **95%**  **KDE (m^2^)** | **50%**  **PHRE (m^2^)** | **95%**  **PHRE (m^2^)** |
| ♂ **1** | Pre | 25 | 41.86 | 184.43 | 24.98 | 162.04 |  |  |
|  | Post | 47 | 1278.83 | 23374.45 | 1640.15 | 10450.81 | 1137.55 | 7851.78 |
| ♀ **2** | Pre | 19 | 7.23 | 10.21 |  |  |  |  |
|  | Post | 15 | 957.95 | 3957.08 |  |  |  |  |
| ♀ **3** | Pre | 19 | 118.31 | 688.97 |  |  |  |  |
|  | Post | 44 | 400.52 | 3753.89 | 1071.42 | 4570.48 | 520.31 | 2846.33 |
| ♂ **4** | Pre | 25 | 40.07 | 485.79 | 70.07 | 517.94 |  |  |
|  | Post | 9 | 404.03 | 499.36 |  |  |  |  |
| ♂ **5** | Pre | 15 | 85.05 | 1263.2 |  |  |  |  |
| ♀ **6** | Pre | 10 | 19.92 | 64.4 |  |  |  |  |
| ♀ **7** | Pre | 26 | 28.38 | 237.39 | 78.82 | 346.13 |  |  |
|  | Post | 48 | 1131.28 | 12151.36 | 2228.06 | 9159.31 | 1173.11 | 6502.08 |
| ♂ **8** | Pre | 26 | 40.54 | 244.03 | 47.33 | 354.28 |  |  |
|  | Post | 23 | 1321.44 | 19645.33 | 7208.37 | 25401.53 | 2597.63 | 13836.53 |
| ♀ **9** | Pre | 15 | 65.42 | 755.68 |  |  |  |  |
| ♀ **10** | Pre | 24 | 5.24 | 2.84 | 0.31 | 1.95 |  |  |
|  | Post | 24 | 746.69 | 4973.64 | 1104.1 | 12705.03 | 1519.85 | 10642.39 |
| ♂ **11** | Pre | 33 | 18.02 | 118.73 | 3.9 | 39.2 |  |  |
| ♀ **12** | Pre | 13 | 16.13 | 96.22 |  |  |  |  |
| ♂ **13** | Pre | 23 | 24.58 | 53.33 | 2.27 | 20.99 |  |  |
|  | Post | 22 | 641.65 | 9520.22 | 2833.97 | 8701.14 | 988.27 | 5286.95 |
| ♀ **14** | Pre | 14 | 30.90 | 223.99 |  |  |  |  |
|  | Post | 64 | 236.13 | 2840.3 | 304.11 | 2670.96 | 353.51 | 2301.83 |
| ♂ **15** | Pre | 15 | 36.74 | 223.31 |  |  |  |  |
|  | Post | 64 | 329.64 | 2115.71 | 302.67 | 1849.94 | 559.72 | 2533.43 |
| ♀ **16** | Pre | 15 | 56.54 | 190.63 |  |  |  |  |
|  | Post | 46 | 472.11 | 3492.24 | 1261.28 | 6143.2 | 676.86 | 3572.12 |
| ♂ **17** | Pre | 12 | 21.51 | 25.08 |  |  |  |  |
|  | Post | 40 | 407.21 | 1923.55 | 319.18 | 1547.26 | 268.19 | 1371.99 |
