# Supplementary material for: Evaluating translocation success of wild eastern hellbenders (Cryptobranchus alleganiensis alleganiensis) in Blue Ridge Ecoregion streams using pre- and post-translocation home range sizes and movement metrics
Source: PLoS One. 2023 Apr 20;18(4):e0283377. doi: 10.1371/journal.pone.0283377 (PMC10118149; doi:10.1371/journal.pone.0283377)
Supplement: S6 Table — Parameter estimates for the top-ranked models describing linear home ranges (LHRs) for wild translocated Cryptobranchus a. alleganiensis in eastern TN, USA, 2018–2019. * = Confidence interval does not overlap zero. (DOCX) [file pone.0283377.s011.docx]

## Table S6. LHR Parameter Estimates.

Parameter estimates for the top-ranked models describing linear home ranges (LHRs) for wild translocated *Cryptobranchus a. alleganiensis* in eastern TN, USA, 2018-2019.

* = Confidence interval does not overlap zero.

| Model | Parameter | Estimate | Std. Error | Lower 95% CI | Upper 95% CI |
| --- | --- | --- | --- | --- | --- |
| LHR – (Trans. inds.) |  |  |  |  |  |
| Nested Rock Size | (Intercept) | 0.0035214 | 0.0009990 | 0.00195 | 0.006 |
|  | *Cohort-2 | -0.0018335 | 0.0010453 | -0.00431 | -0.0001 |
|  | *Cohort-1:R_SIZE_ | 0.0044246 | 0.0022668 | 0.00041 | 0.0095 |
|  | Cohort-2: R_SIZE_ | 0.0004846 | 0.0002708 | -0.000038 | 0.001 |
| Cohort | (Intercept) | 0.0045 | 0.0013 | 0.0025 | 0.0074 |
|  | *Cohort-2 | -0.003063 | 0.0013 | -0.006 | -0.00094 |
| Environment + Cohort | (Intercept) | 0.0042 | 0.0012 | 0.0022 | 0.0071 |
|  | R_SIZE_ | 0.00057 | 0.000302 | -0.00003.17 | 0.001 |
|  | R_DENS_ | 0.000046 | 0.000257 | -0.000401 | 0.00061 |
|  | *Cohort-2 | -0.0025 | 0.0013 | -0.0054 | -0.00028 |
