# Supplementary material for: Evaluating translocation success of wild eastern hellbenders (Cryptobranchus alleganiensis alleganiensis) in Blue Ridge Ecoregion streams using pre- and post-translocation home range sizes and movement metrics
Source: PLoS One. 2023 Apr 20;18(4):e0283377. doi: 10.1371/journal.pone.0283377 (PMC10118149; doi:10.1371/journal.pone.0283377)
Supplement: S7 Table — Validation statistics for top models (see Table 5) of each modeling analysis for predicting sedentariness and linear home ranges (LHR) of Eastern Hellbenders (Cryptobranchus a. alleganiensis) using k-fold cross-validation. Sedent. = Sedentariness. Values that are not given as a percentage here (i.e. not AB % or RE %), are in the units of the response variable (i.e. as a proportion between 0–1 for sedentariness, and meters for LHR). Formulas for these metrics (and brief explanations) are given in S2 Appendix. *—Model-averaging used for predictions. (DOCX) [file pone.0283377.s012.docx]

## Table S7. Top Model Validation Statistics.

Validation statistics for top models (see Table 5) of each modeling analysis for predicting sedentariness and linear home ranges (LHR) of Eastern Hellbenders (*Cryptobranchus a. alleganiensis*) using *k*-fold cross-validation. Sedent. = Sedentariness. Values that are not given as a percentage here (i.e. not AB % or RE %), are in the units of the response variable (i.e. as a proportion between 0-1 for sedentariness, and meters for LHR). Formulas for these metrics (and brief explanations) are given in Appendix B. * - Model-averaging used for predictions

| Validation Statistics | Sedent. –  All Inds. | Sedent. – Trans. Inds. | LHR –  All Inds. | *LHR –  Trans. Inds. |
| --- | --- | --- | --- | --- |
| Average Bias (AB) | 0.007 | 0.02 | 33.44 | 2.89 |
| Mean Absolute Error (MAE) | 0.11 | 0.08 | 197.4 | 253.3 |
| Prediction Root Mean Square Error (PRMSE) | 0.14 | 0.10 | 410.78 | 304.7 |
| Average Bias Percent (AB %) | 1.27 | 3.04 | 12.25 | 0.54 |
| Relative Error Percent (RE %) | 24.4 | 17.1 | 150 | 58.5 |
| Model Efficiency (EF) | 0.27 | 0.71 | -0.22 | 0.29 |
