# Supplementary material for: Evaluating translocation success of wild eastern hellbenders (Cryptobranchus alleganiensis alleganiensis) in Blue Ridge Ecoregion streams using pre- and post-translocation home range sizes and movement metrics
Source: PLoS One. 2023 Apr 20;18(4):e0283377. doi: 10.1371/journal.pone.0283377 (PMC10118149; doi:10.1371/journal.pone.0283377)
Supplement: S1 Appendix — (DOCX) [file pone.0283377.s013.docx]

# S1 Appendix

**Weighting Formula**

The following formula was used for weighting and averaging way points taken from the same location. The weight assigned to a number to be averaged was calculated based on the area (A) of overlap between a circle with a radius (R) of the greatest possible accuracy of the unit (e.g. 3 meters) and a circle with a radius (r) of the actual reported accuracy, which in this case also represents the furthest distance (d; d = r) that the given GPS coordinate could be from the “true” coordinate at that location, given by the formula:

$$A\left( d;R,r \right)=\beta R^{2}+\alpha r^{2}-\frac{1}{2}r^{2}\sin2\alpha-\frac{1}{2}R^{2}\sin2\beta,$$

where

$\cos\alpha= \frac{r^{2}+ d^{2}- R^{2}}{2rd}$ and $\cos\beta= \frac{R^{2}+d^{2}-r^{2}}{2Rd}$ .
