# Supplementary material for: Evaluating translocation success of wild eastern hellbenders (Cryptobranchus alleganiensis alleganiensis) in Blue Ridge Ecoregion streams using pre- and post-translocation home range sizes and movement metrics
Source: PLoS One. 2023 Apr 20;18(4):e0283377. doi: 10.1371/journal.pone.0283377 (PMC10118149; doi:10.1371/journal.pone.0283377)
Supplement: S2 Appendix — (DOCX) [file pone.0283377.s014.docx]

# S2 Appendix

**Equations for Model Validation Metrics**

Model prediction errors for each model were estimated using Equation 1, by calculating differences between the observed and model-predicted values.

1. $e_{i}=\left( Y_{i}-\hat{Y}_{i} \right)$

Where $e_{i}$ is error, $Y_{i}$ is observed value, and $\hat{Y}_{i}$ is predicted value. Model validation statistics were subsequently calculated using the equations below. A value of an average bias of zero would indicate no bias in the model, while a positive value indicates under-prediction by the model and a negative value indicates over-prediction. Average bias percent provides a relative metric of the level of bias from the model predictions.

1. Average Bias $\left( \bar{e} \right)= \frac{\sum_{i=1}^{n} \left( Y_{i}- \hat{Y}_{i} \right)}{n}$
2. Average Bias Percent $\left( \bar{e} \% \right)= \frac{\sum_{i=1}^{n} \left( Y_{i}- \hat{Y}_{i} \right)}{\sum_{i=1}^{n} \left( Y_{i} \right)} \times100$

Mean absolute error (MAE) and Prediction root mean square error (PRMSE) are used to indicate model prediction uncertainties. Relative error percent (RE%) is used to describe the relative size of model prediction uncertainty.

1. Mean Absolute Error (MAE) $= \frac{\sum_{i=1}^{n} \left( \left| Y_{i}- \hat{Y}_{i} \right| \right)}{n}$
2. Prediction Root Mean Square Error (PRMSE) $= \sqrt{\frac{\sum_{i=1}^{n} \left( Y_{i}- \hat{Y}_{i} \right)^{2}}{n}}$
3. Relative Error Percent (RE%) $= \frac{\sqrt{\sum_{i=1}^{n} \frac{\left( Y_{i}- \hat{Y}_{i} \right)^{2}}{n}}}{\sum_{i=1}^{n} \left( Y_{i} \right)/n} \times100$

Lastly, Model efficiency (EF) is used to indicate an overall goodness of fit, by comparing the predictions to the mean of all observed values, or $\bar{Y}$. As described by Pinjuv et al. (2006), a value close to 1 indicates a near perfect fit, while a value above zero indicates the predictions are better than the average, and a negative value indicates the model is a poor fit.

1. Model Efficiency (EF) $=1- \frac{\sum_{i=1}^{n} \left( Y_{i}- \hat{Y}_{i} \right)^{2}}{\sum_{i=1}^{n} \left( Y_{i}- \bar{Y} \right)^{2}}$
